# Discrimination and Quantitation of Biologically Relevant Carboxylate Anions Using A [Dye•PAMAM] Complex

**DOI:** 10.3390/s21113637

**Published:** 2021-05-24

**Authors:** Yifei Xu, Marco Bonizzoni

**Affiliations:** 1 Department of Chemistry and Biochemistry, The University of Alabama, Tuscaloosa, AL 35487, USA; xxu56@crimson.ua.edu; 2 Alabama Water Institute, The University of Alabama, Tuscaloosa, AL 35487, USA

**Keywords:** carboxylate, sensing, pattern, fluorescence, dendrimer, quantitation

## Abstract

Carboxylate anions are analytical targets with environmental and biological relevance, whose detection is often challenging in aqueous solutions. We describe a method for discrimination and quantitation of carboxylates in water buffered to pH 7.4 based on their differential interaction with a supramolecular fluorescent sensor, self-assembled from readily available building blocks. A fifth-generation poly(amidoamine) dendrimer (PAMAM G5), bound to organic fluorophores (calcein or pyranine) through noncovalent interactions, forms a [dye•PAMAM] complex responsive to interaction with carboxylates. The observed changes in absorbance, and in fluorescence emission and anisotropy, were interpreted through linear discriminant analysis (LDA) and principal component analysis (PCA) to differentiate 10 structurally similar carboxylates with a limit of discrimination around 100 μM. The relationship between the analytes’ chemical structures and the system’s response was also elucidated. This insight allowed us to extend the system’s capabilities to the simultaneous identification of the nature and concentration of unknown analytes, with excellent structural identification results and good concentration recovery, an uncommon feat for a pattern-based sensing system.

## 1. Introduction

**Scheme 1:**
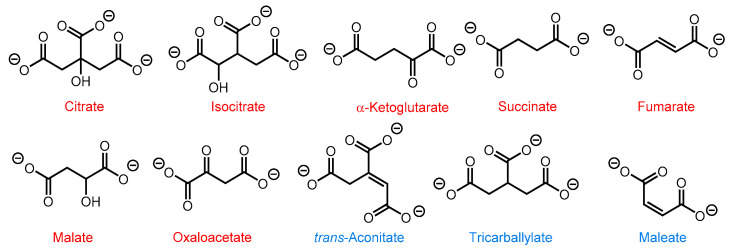
Structures of the carboxylates of interest (red: citric acid cycle intermediates, blue: other, structurally similar carboxylates), shown in their protonation state in water at pH 7.4; see Table S1 for relevant p*K_a_* values.

Carboxylates can be recognized using complex biomolecules as sensors with high selectivity [1,2], but synthetic chromogenic or fluorogenic chemosensors have garnered interest for their high sensitivity, ruggedness, and fast operation [3]. Supramolecular approaches to anion sensing using non-covalent sensing complexes have also proved versatile and easy to implement [4,5,6]. Synthetic sensors, however, are often restricted to aprotic solvents [7,8], precluding biochemical or environmental applications. Here, we report on a supramolecular sensing system for the qualitative and quantitative discrimination of carboxylates that works in water buffered to pH 7.4. These are desirable but challenging conditions, because the strong interactions of water-soluble anions with water are difficult to overcome in synthetic systems. As a proof of viability, we selected seven carboxylates involved in the citric acid cycle as analytes (shown in red in Scheme 1), as well as three structurally similar carboxylates (shown in blue) also commonly found in physiological media that may act as interferents. Not only are these anions involved in the energy machinery of aerobic cells; some of them also participate in biological signaling [9,10], and mis-regulation of their metabolism is linked to multiple diseases [11]. Citrate can also be used as a reliable screening target for prostate cancer [12,13]: in fact, normal prostatic fluid contains high levels of citrate (400–1500 times higher than in plasma); however, premalignant and malignant prostate cells convert most of their citrate to isocitrate, so their citrate level is far lower than normal [14]. Existing sensors for citrate do not display good selectivity between citrate and isocitrate [15,16]. Differentiation of isomers is also important for fumarate, whose stereoisomer maleate causes kidney diseases (e.g., Fanconi syndrome) [17]. While, in the latter case, selective sensing of either stereoisomer has been reported [18,19], a comprehensive approach to the discrimination of carboxylates is still missing.

Our group has used poly(amidoamine) (PAMAM) dendrimers as supramolecular hosts for anionic organic compounds in array-based sensing applications with high selectivity using simple instrumentation and commercially available building blocks [20,21]. PAMAM dendrimers are water soluble globular hyperbranched polymers whose surface is terminated in a dense array of primary amine groups, displaying a high binding capacity for smaller organics through non-covalent interactions [22,23].

Their surface carries positive charges in water at neutral pH, due to protonation of roughly half the surface amine groups [24], so they can establish hydrogen bonding and electrostatic interactions with anionic species. Here, we harness these interactions for sensing and discrimination of carboxylate analytes using chemical fingerprinting methods in water buffered to pH 7.4, and we extend these results to achieve simultaneous determination of identity and concentration of these analytes.

## 2. Materials and Methods

Fifth-generation amine-terminated PAMAM dendrimers with 1,2-diaminoethane core were purchased from Dendritech (Midland, MI, USA) as 4.99% solution in MeOH. Calcein was purchased from Sigma Aldrich (St. Louis, MO, USA) and pyranine from Alfa Aesar (Haverhill, MA, USA). Carboxylic acids were purchased from Sigma Aldrich, Alfa Aesar, Acros Organics (Geel, Belgium), and EMD Millipore (Burlington, MA, USA). All materials were used as received. All working solutions were prepared in DI water, buffered to pH 7.4 with 50 mM 2-[4-(2-hydroxyethyl)piperazin-1-yl]ethanesulfonic acid (HEPES).

Benchtop titrations were carried out in 1-cm square quartz cuvettes on a HP 8452A diode array spectrophotometer (for absorbance), or on an ISS PC1 spectrofluorometer (for fluorescence emission and anisotropy). Sample temperature was controlled by an external circulating water bath. A Biotek Synergy II multimode plate reader was used for multivariate studies in Greiner BioOne non-treated black-wall polystyrene 384-well plates with clear bottoms. The total volume in each well was 100 µL. A complete list of wavelengths and conditions used in the multivariate data acquisition is reported in the Supporting Information. Data reduction and analysis was carried out in Wolfram Mathematica v. 12.0, using code developed in-house.

For concentration determination, two standard curves were made by averaging the 4 tricarboxylates’ and 6 dicarboxylates’ fluorescence anisotropy profiles, respectively. Solutions of randomly chosen carboxylates were prepared by a colleague, Dr. Xiaoli Liang, as “unknown samples”; the known concentration of these samples was not disclosed to us until after the conclusion of our experiments. Their pH was adjusted to pH 7.4 and titrations were performed for each unknown sample into [calcein•PAMAM]. Fluorescence anisotropy readings were compared to the standard curves to calculate two hypothetical concentrations, for the tricarboxylate or dicarboxylate case, respectively; the samples were then diluted to 2.5 mM based on the calculated concentrations, and deposited on a 384-well plate which also contained carboxylate reference samples at the same concentration. Data analysis was carried out, as described above and in the Supporting Information.

## 3. Results

### 3.1. Binding of Dye to PAMAM G5 Dendrimer

**Scheme 2:**
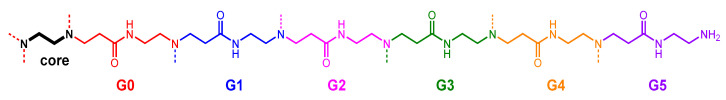
One branch of a 5th generation (G5) poly(amidoamine) (PAMAM) dendrimer, amine-terminated, and with an 1,2-diaminoethane core. The dashed lines indicate the position of another branch along the structure.

**Scheme 3:**
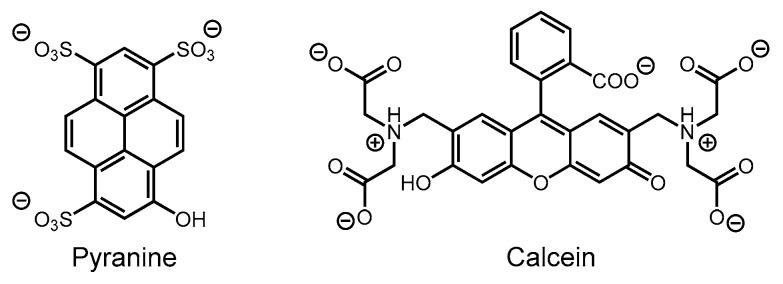
Structures of calcein and pyranine in their protonation state in water buffered to pH 7.4; see Table S1 for p*K_a_* values.

In this work 5th generation (G5) amine-terminated PAMAM dendrimers were used (Scheme 2) because they provide optimal balance between high surface group density and affordability. All work was carried out in water, buffered to pH 7.4 with 50 mM aqueous HEPES buffer. We selected optical spectroscopic techniques (absorbance and fluorescence) to monitor the binding between PAMAM dendrimers and carboxylates because of their high sensitivity, ubiquity, and ease of use. However, neither the PAMAM receptors nor the carboxylates have detectable signals in the visible region of the spectrum. We circumvented this problem by building a dye displacement assay to transform these optically silent receptors into chemical sensors. Two organic dyes, calcein and pyranine (Scheme 3), were chosen after screening (see ESI, Scheme S1) to form a [dye•PAMAM] sensing complex because of their high quantum yield, anionic charge, stability in water, and commercial availability in good purity. Upon binding of carboxylates to the sensing complex, the dye was displaced; carboxylate binding could then be monitored through the associated changes in the dye’s spectroscopic signal. Analytical discriminatory power was achieved using pattern recognition algorithms such as principal component analysis (PCA) and linear discriminant analysis (LDA) to analyze the raw experimental results [25,26].

#### 3.1.1. Binding of Pyranine

**Figure 1:**
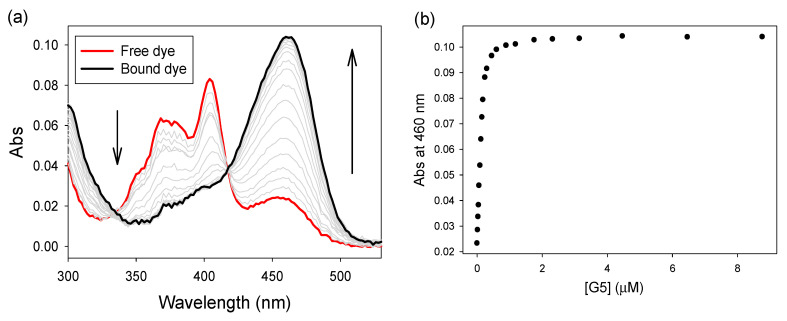
Binding of pyranine to PAMAM G5 dendrimer. (**a**) Absorbance spectra; (**b**) absorbance profile at 460 nm. [pyranine] = 6.04 μM.

On addition of PAMAM G5 into a pyranine solution, formation of the [pyranine•PAMAM] complex was indicated by the appearance of a new, lower energy absorbance band in the absorbance spectrum, with a clear isosbestic point at 417 nm (Figure 1a). With a total pyranine concentration of 6.04 μM, the absorbance profile for this new band at 460 nm (Figure 1b) reached a plateau at G5 concentration around 1 μM, indicating that multiple molecules of dye bound to each PAMAM. In addition to electrostatic and hydrogen bonding interactions between pyranine’s sulfonates and the ammonium groups of PAMAM G5, the complexation may also be aided by hydrophobic interactions between the pyrene core of this dye and the interior of PAMAM G5.

**Figure 2:**
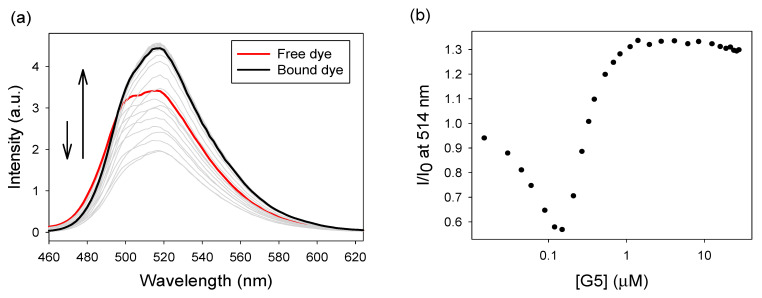
Binding of pyranine to PAMAM G5 dendrimer. (**a**) Fluorescence emission spectra; (**b**) emission intensity profile at 514 nm. [pyranine] = 6.04 μM.

Fluorescence measurements performed in the same conditions (Figure 2a) showed an initial decrease in emission followed by an increase to a plateau (see Figure 2b). When only small amounts of PAMAM G5 are added and the pyranine/G5 ratio is high, a complex is formed with the maximum number of pyranine molecules that can be accommodated per G5 dendrimer. Here, the bound dye molecules are close enough to self-quench by resonance energy transfer (RET). Further additions of dendrimer then decrease the pyranine/G5 ratio, which favors the formation of a 1:1 dye:dendrimer complex, with the dyes well separated; this gradual inactivation of the RET self-quenching results in the observed increase in emission intensity. In this case, the dye was more fluorescent in its bound state than free in solution; increased rigidity in the bound conformation provided fewer opportunities for non-radiative decay.

**Figure 3:**
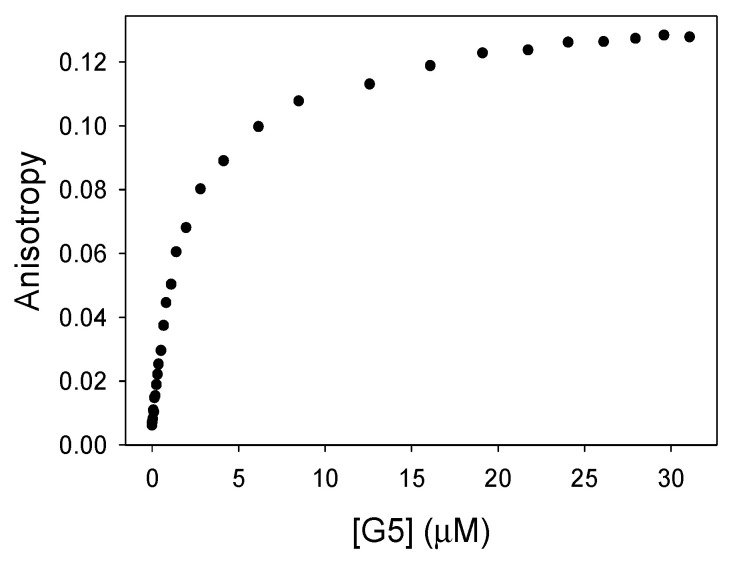
Fluorescence anisotropy profile for the binding of pyranine to PAMAM G5. Excitation: 418 nm; emission: 514 nm. [pyranine] = 6.04 μM.

The anisotropy in the polarization of the fluorescence emission (fluorescence anisotropy for short, Figure 3) was also an excellent reporter of binding because its increase can only be due to the restriction of the small dye’s tumbling motion caused by binding to the much larger PAMAM G5. The equivalence point of this titration appears higher, but that is caused by RET scrambling of the anisotropy, a known side effect of the RET quenching mentioned above [27,28].

#### 3.1.2. Binding of Calcein

**Figure 4:**
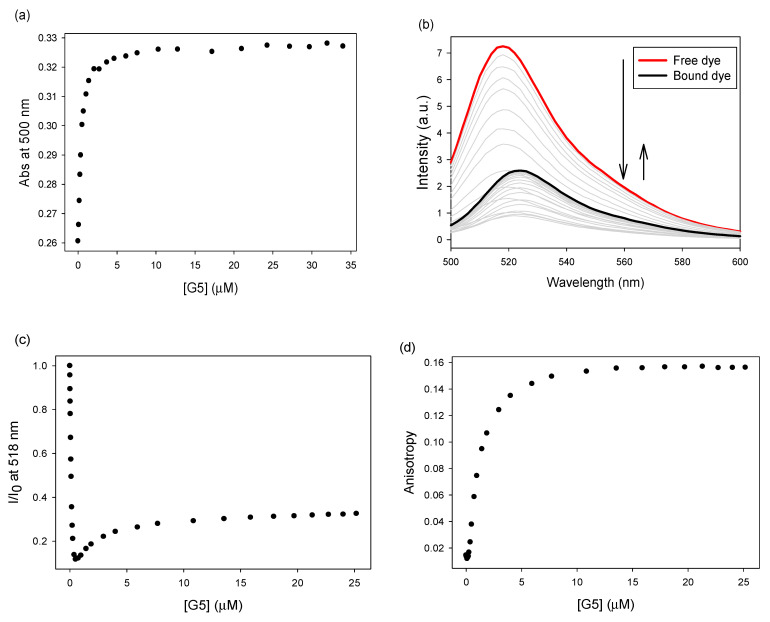
Binding of calcein to PAMAM G5 dendrimer. (**a**) Absorbance profile at 500 nm; (**b**) fluorescence emission spectra; (**c**) fluorescence emission profile; and (**d**) fluorescence anisotropy. Excitation: 496 nm; emission: 518 nm. [calcein] = 6.36 μM.

Calcein, the second dye we selected, also showed good affinity for PAMAM G5: binding profiles are reported in Figure 4 and spectra in Figure S1 in the ESI. The binding to PAMAM G5 caused a red shift in calcein’s absorbance peak and a fluorescence emission trend similar to that observed for pyranine. In this case, however, the bound dye had lower emission intensity than the free dye; we previously reported this behavior for the structurally related fluorescein, which is also a xanthene dye, and attributed it to reductive quenching by the dendrimer’s free amine groups [29,30]. The amount of G5 required to reach saturation was higher for calcein than for pyranine, suggesting that pyranine binds to PAMAM G5 more strongly than calcein. This cannot stem from the electrostatic inter-actions (they are both trianions); instead, it is likely that the more hydrophilic calcein establishes fewer favorable hydrophobic interaction with the dendrimer [31]. This already unfavorable situation is exacerbated by calcein’s higher energetic cost of de-solvation upon binding: carboxylate groups in calcein are better H bonding acceptors than the sulfonate groups in pyranine, therefore calcein is likely to form stronger hydrogen bonding interactions with water molecules compared to pyranine and to be better solvated in water. On the other hand, we believe that differences in size or flexibility between the two dyes do not play a significant role in determining their relative affinities: in fact, previous work in this group found little difference in binding affinity between flexible and rigid carboxylates with the same charge towards amine-terminated PAMAM dendrimers [20].

### 3.2. Binding of Carboxylate to PAMAM G5 Dendrimer (Indicator Displacement Assay)

Once we established that pyranine and calcein have strong affinity to G5, and that their binding to the dendrimer is accompanied by easily detectable changes in their optical signal, we set up an indicator displacement assay (IDA) to detect the binding of the carboxylates of interest to the G5 PAMAM dendrimer. In our experience conditions where ca. 85% of the indicator dye is bound to the receptor are ideal for an IDA [32]; such conditions were found for each dye by inspection of the binding profiles presented above (namely, [pyranine] = 6.04 μM + [G5] = 0.213 μM; [calcein] = 6.36 μM + [G5] = 2.13 μM).

#### 3.2.1. A Pyranine-Based IDA

**Figure 5:**
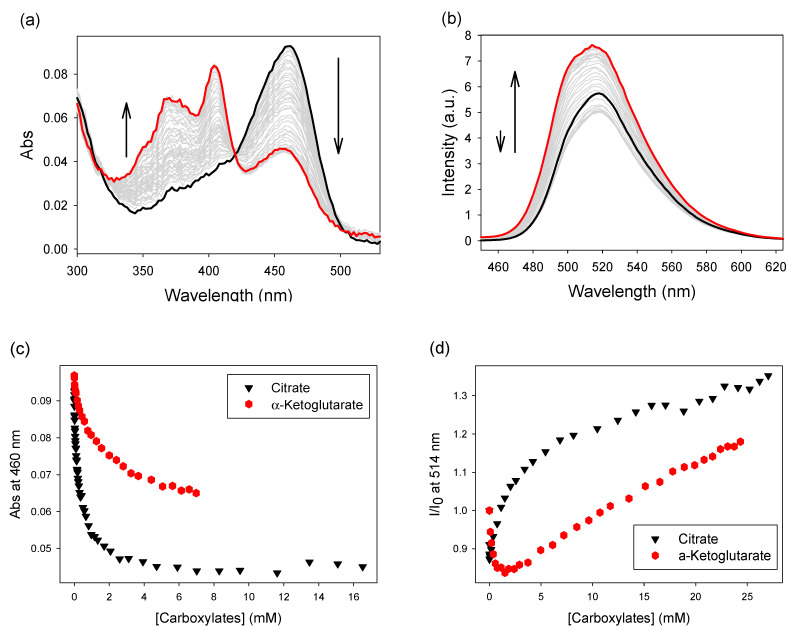
Binding of carboxylates to PAMAM G5 using pyranine as indicator. Titration of citrate into [pyranine•PAMAM] (the black spectrum represents the titration’s starting point; the red one represents the last point): (**a**) absorbance spectra; (**b**) fluorescence emission spectra. Titration profiles for binding citrate and α-ketoglutarate to PAMAM G5: (**c**) absorbance; (**d**) fluorescence emission. Excitation: 420 nm, emission: 514 nm. [pyranine] = 6.04 μM, [G5] = 0.213 μM.

Figure 5a,b shows the absorbance and fluorescence emission spectra obtained during titration of a solution containing the [pyranine•PAMAM] sensing complex with citrate. In the absorption spectra, the intensity of the free pyranine absorption band increased (400 nm), whereas the absorption of the complex decreased (460 nm). This behavior is precisely the opposite of that observed upon binding of the dye to PAMAM (compare to Figure 1). The reversal in the spectral features of the solution indicates that the opposite process to dye binding is taking place, i.e., the dye is being released from its complex with PAMAM; this occurs because the binding sites on PAMAM G5 are being occupied by the citrate titrant, indicating that citrate was binding to G5. The fluorescence emission signal showed a similar inversion (Figure 5d), confirming citrate binding.

Studying a second probe anion, α-ketoglutarate, confirmed the generality of this approach. Figure 5c,d shows the absorbance and fluorescence emission profiles for citrate and α-ketoglutarate when interacting with the [pyranine•PAMAM] complex (see ESI, Figure S2 for corresponding absorbance and fluorescence spectra), indicating successful binding of this second anion. Although the general trends are similar between the two anions, it should be noted here that there are clear differences: in the following, we will harness these to effect carboxylate differentiation.

#### 3.2.2. A Calcein-Based IDA

**Figure 6:**
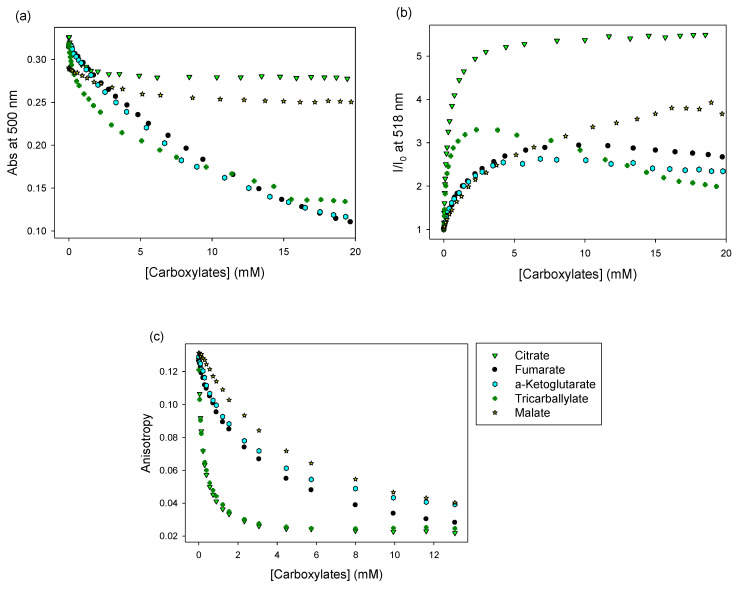
Carboxylate binding to PAMAM G5, monitored by calcein displacement. Profiles: (**a**): absorbance at 500 nm; (**b**): fluorescence emission at 518 nm; (**c**) fluorescence anisotropy at 518 nm. Excitation: 496 nm or 486 nm depending on the isosbestic point (see ESI for detail). [calcein] = 6.36 μM, [G5] = 2.13 μM.

We expanded on these results by using calcein to form a [calcein•PAMAM] sensing complex, and more probe anions (citrate, fumarate, α-ketoglutarate, tricarballylate, and malate). The resulting absorbance and fluorescence spectra are included in Figure S3 in the ESI; the profiles are shown in Figure 6 below. Absorbance and fluorescence showed a clear inversion of the trends observed for dye binding. The fluorescence emission increased monotonically on addition of anions as the freed-up calcein went back to the bulk solution, where its fluorescence is higher.

Fluorescence anisotropy was once again the most direct indicator of binding; on addition of carboxylates it smoothly decreased to the low value typical of the free dye, indicating dye displacement and carboxylate binding to PAMAM G5 (Figure 6c). The slope of each anion’s anisotropy titration profile is proportional to its affinity for PAMAM G5; as expected, higher-charged anions (e.g., tricarboxylates) were found to have higher affinity to the polycationic PAMAM G5 [20].

### 3.3. Anion Discrimination

As previously mentioned, the carboxylates studied showed differential behavior on binding to PAMAM G5, but the differences were too subtle for direct interpretation, so we set up a pattern-based chemical recognition system, using the two [PAMAM•dye] complexes as sensors, and exposing both dye–dendrimer complexes to each carboxylate anion in our analyte panel (Scheme 1). The analysis was carried out on a 384-well plate; a microwell plate reader was used for absorbance and fluorescence detection providing fast automated reading of multiple variables (absorbance, fluorescence emission and anisotropy). Using a 384-well plate also allowed us to run each sample in multiple replicates on the same plate; the small well volume also minimized reagent consumption and worked with smaller analytical samples.

The carboxylate anion samples were deposited at the optimal discrimination concentration determined from the binding profiles as discussed above, i.e., [carboxylates] = 2.04 mM for pyranine, and 2.30 mM for calcein. These concentrations are high enough that differences in behavior between our sample anions were significant, but low enough to make the method analytically valuable.

Multiple optical measurements were collected for each sample on the plate, generating a multi-dimensional dataset that was further processed through linear discriminant analysis (LDA) and principal component analysis (PCA) to extract useful information towards carboxylate discrimination. In a first step, invariant or highly correlated raw instrumental measurement (“variables”) were identified and removed from the dataset because they would contribute no discriminatory power but decrease the overall signal-to-noise ratio; further preliminary screening details, including outlier rejection, are summarized in the ESI (Figure S4). The dataset was then subjected to LDA and PCA algorithms. Both techniques transformed the original dataset (i.e., the raw measurements) into a new one with the same dimensionality (factors or principal components, for LDA and PCA respectively), obtained as appropriately chosen linear combinations of the raw measurements. Critically, the factors or principal components are returned in order of decreasing information content. The transformed data set contained the same information as the original one, but “concentrated” in fewer descriptor variables; however, it was still high-dimensional, so data reduction was necessary for presentation. This was accomplished by retaining only the first two factors or principal components in each case, typically with minimal loss of information. After data reduction, each sample was represented by two scores, i.e., their coordinate along each one of the two new descriptor variables generated by the analysis algorithm, which located each sample on a 2D scatter plot, the “scores plot”. The corresponding “loadings plot” provided the relative contribution of each raw instrumental variable to the new descriptors, allowing us to link the results of this analysis to chemical or structural features of the analytes.

#### 3.3.1. Using the Pyranine-Based Sensor

**Figure 7:**
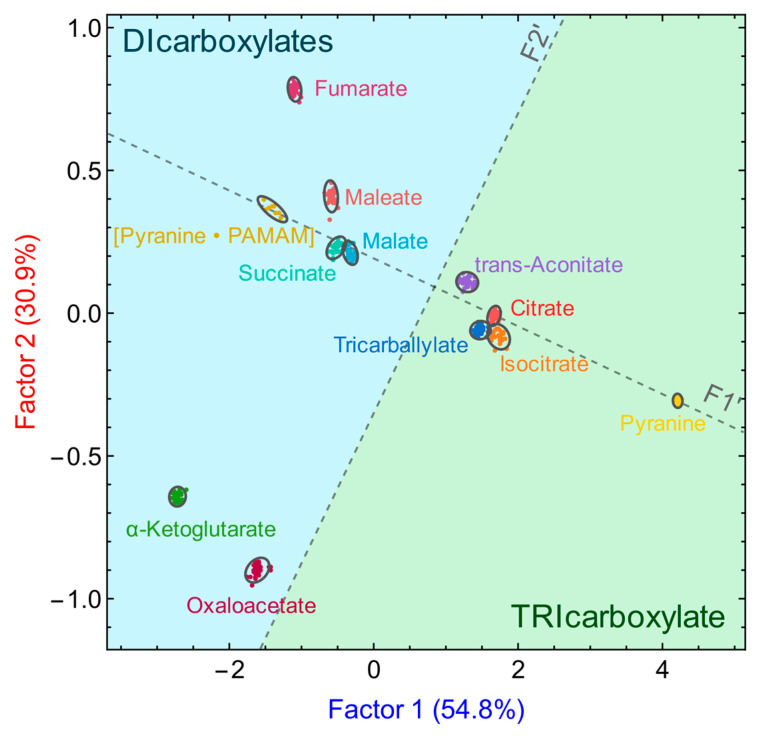
LDA scores plot for the discrimination of 10 carboxylates (2 mM) using the [pyranine•PAMAM] complex sensor. Blue region: dicarboxylate clusters, green region: tricarboxylate clusters. [pyranine] = 6.04 μM, [PAMAM G5] = 0.213 μM, [carboxylates] = 2.04 mM. Performed in 50 mM aqueous HEPES buffer at pH 7.4, T = 25 °C.

The [pyranine•PAMAM] sensor was exposed to samples of the carboxylate analytes (Scheme 1) at the optimal discrimination concentration identified for pyranine (2.04 mM); 38 instrumental measurements (absorbances, fluorescence emission intensities at various wavelengths) were then acquired. After data analysis, the resulting LDA scores plot is shown in Figure 7. Factor 1 summarizes 54.8% of the information contained in the original dataset, factor 2 another 30.9%; in total, 85.7% of the original information was retained in this scores plot, even after drastic data reduction from a 38-dimensional data set. The information distribution between the two factors was also well balanced, indicating that multiple independent factors contribute significantly to the differentiation, and validating the use of multivariate analysis. The clusters of replicates are reasonably small compared to the average intercluster distance, although this differentiation attempt using pyranine was still incomplete: there was some overlap among clusters, so samples were not fully differentiated. One such example is the citrate-isocitrate-tricarballylate supercluster: these structurally similar trianionic carboxylates could not yet be completely differentiated here.

Even though discrimination was still incomplete, the relative position of the sample clusters is easily linked with the samples’ chemical structure. Two larger groupings (“superclusters”) can be identified in the scores plot, the one mentioned for citrate, isocitrate, and tricarballylate, and another for malate and succinate. The anions in each supercluster are structurally very similar, the only difference being the presence of hydroxy groups in some structures (see Scheme 1). Unfortunately, in the case of the [pyranine•PAMAM] complex the presence or absence of an OH group did not provide enough chemical diversity to affect the separation.

Furthermore, considering a convenience axis F1′ that connects the free dye reference cluster to the bound dye in the scores plot, the projections of all cluster centroids onto this axis can be correlated to the affinity of the respective anions for the PAMAM G5 dendritic host. In fact, in this system the total stoichiometric concentration is the same for all anions, so the extent of dye displacement reflects the extent of the anion’s own binding which, in turn, is controlled by their relative affinity to the PAMAM receptor. Tricarboxylates, which have higher affinity for the G5 polycations [20], resulted in a more complete displacement of the dye; therefore, in a tricarboxylate–PAMAM–dye solution, the dye was mostly found in its free state, and these samples appeared spectroscopically similar to the free dye, so their clusters were found closer to the “free dye” reference along the F1′ axis in the scores plot. Similar reasoning explains the fact that samples of the lower-affinity dicarboxylate anions, which contain a larger portion of PAMAM-bound dye, ended up closer to the “bound dye” reference.

Furthermore, scores along a second ancillary axis F2′, running through the center of the scores plot and perpendicular to F1′, correlated well with the strength of the corresponding anions’ absorption in the UV range. In fact, oxaloacetate and α-ketoglutarate, which contain a C=O group and have the highest intrinsic UV absorption among the anions considered, ended up with the largest absolute scores along F2′, followed by fumarate, maleate and *trans*-aconitate that contain the weaker C=C chromophore. In further support of this hypothesis, the clusters lying directly on F1′ (i.e., with near zero score on F2′) correspond to citrate, malate, and succinate, which have no UV absorption.

Finally, the LDA loadings for this analysis (Figure S6, Table S2 in the ESI) indicate that differentiation along factor 1 was driven by differences in absorbance among the various analytes in their interaction with the [PAMAM•pyranine] sensing complex, whereas it was mostly differences in fluorescence emission that induced differentiation along factor 2.

The corresponding results from PCA analysis are shown in Figure S5; unfortunately, in this case, many dicarboxylate clusters were overlapped, providing worse differentiation than LDA.

#### 3.3.2. Using the Calcein-Based Sensor

**Figure 8:**
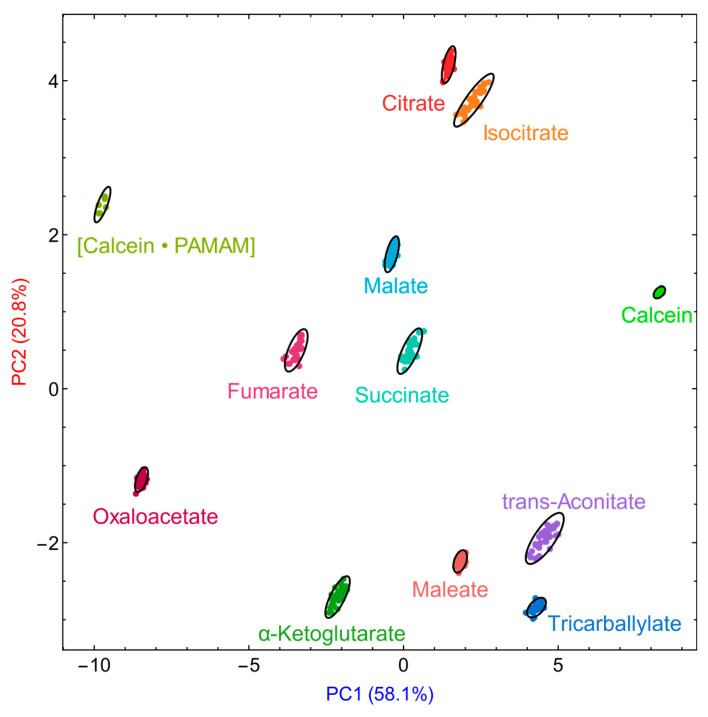
PCA scores plot for the discrimination of the 10 carboxylates using the [calcein•PAMAM] complex sensor. [calcein] = 6.36 μM, [PAMAM G5] = 2.13 μM, [carboxylates] = 2.30 mM. Performed in 50 mM aqueous HEPES buffer at pH 7.4, T = 25 °C.

In this case, we were pleased to see that PCA led to very good separation, as shown in Figure 8 (Figure S7 and Table S3 in the ESI show the corresponding loadings). This was exciting because PCA is a more demanding unsupervised clustering technique (i.e., the identity of the samples is not provided to the algorithm), so its ability to fully separate these samples indicated to us that the calcein-based sensing complex had an intrinsically higher discriminating power than pyranine’s. Indeed, we obtained excellent differentiation using PCA with the calcein sensor, with tight and well separated clusters, overall high information retention (79%), and an excellent balance of contributions between the two principal components, which indicates that the analysis takes full advantage of the available spectroscopic features for discrimination. Unlike LDA, all raw measurements contributed to the differentiation; in particular, a combination of absorbance, fluorescence emission and anisotropy measurements contributed to PC1, whereas only absorbance measurements contributed to PC2.

**Figure 9:**
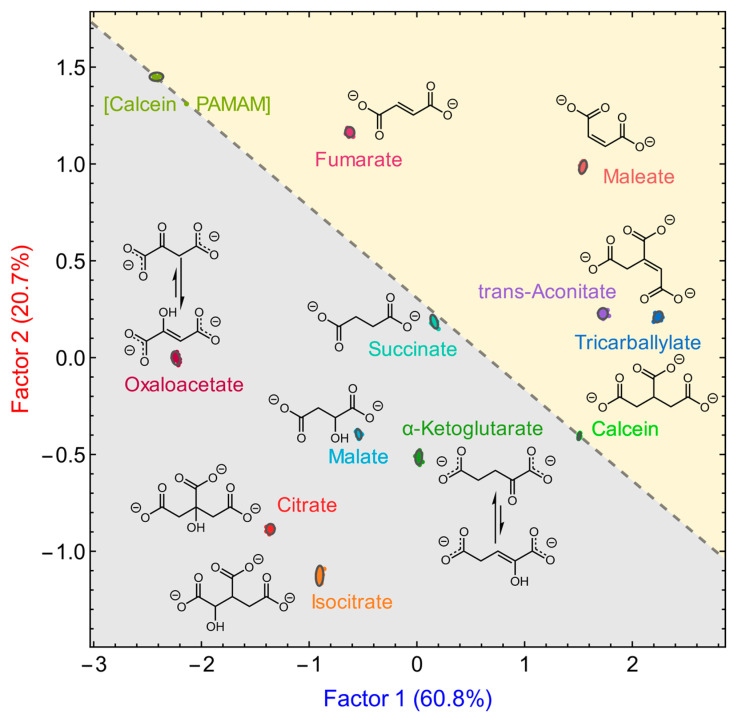
LDA scores plot for the discrimination of the 10 carboxylates using the [calcein•PAMAM] complex sensor. Gray region: hydroxycarboxylate clusters, yellow region: carboxylates that do not contain a hydroxy group. [calcein] = 6.36 μM, [PAMAM G5] = 2.13 μM, [carboxylates] = 2.30 mM. Performed in 50 mM aqueous HEPES buffer at pH 7.4, T = 25 °C.

We performed LDA analysis on this dataset as well. The two-dimensional scores plot obtained after data reduction, is shown in Figure 9, using 29 of the 42 raw measurements, and capturing 81.5% of the total information content in the original dataset. In this case, all carboxylates were well separated, including citrate and isocitrate (the most similar in the panel). The clusters of replicates for the same sample are very tight, indicating excellent reproducibility, whereas intercluster distances are quite large, showcasing the strong discriminatory power of the method. Tight, well separated clusters also imply a higher tolerance against outliers.

Here too we saw a distinction between trianionic and dianionic analytes displaying respectively higher and lower binding affinity; as seen with the pyranine discrimination results, here too the projections of the tricarboxylate clusters on the line connecting the free and bound dye clusters (the dotted line in Figure 9 are closer to the free dye than those of dicarboxylates, further validating our previous line of reasoning.

The separation brought about by the [calcein•PAMAM] sensing complex could be connected to the analytes’ chemical features as well. In fact, the resulting LDA scores plot could be divided in two regions, in grey and yellow in Figure 9. On the one hand, analytes containing hydroxyl functional groups were found in the grey region. Note that, in this respect, oxaloacetate and α-ketoglutarate are categorized as hydroxyacids together with citrate, isocitrate, and malate, because in aqueous solution they are predominantly found in their enol form, as shown in Figure 9. Additionally, the enol form of oxaloacetate can form an effective intramolecular hydrogen bond between the OH group of the enol and the distal carboxylate group, in a six-membered ring interaction; on the other hand, the same H-bond in the enol form of α-ketoglutarate is not as stabilizing because it would form a seven-membered ring. The enol form of oxaloacetate is more stable and therefore more abundant than that of α-ketoglutarate, so oxaloacetate has a more pronounced “hydroxycarboxylate character” than α-ketoglutarate. This causes the oxaloacetate cluster to be found deeper into the hydroxyacid region than α-ketoglutarate and further away from the separator line. On the other hand, carboxylates with other chemical features in addition to the COO^−^ group, but no OH groups, were found in the yellow region: examples of such additional chemical features are, for instance, the C=C bond in fumarate, maleate, and *trans*-aconitate, and the closely spaced and interacting carboxylate groups in maleate, *trans*-aconitate, and tricarballylate. Finally, “featureless” carboxylate such as succinate (1,4-butanedicarboxylate) did not belong to either of these two groupings; instead, their clusters were found close to the “free dye–bound dye” construction line. The importance of the hydroxy feature in driving further differentiation among carboxylates with otherwise similar charge and structure agrees with previous results from our group [20], which indicated that the amine-terminated PAMAM polyelectrolytes act as hydrogen bond acceptors, so they are sensitive to the presence of hydrogen bond donor groups such as OH in their binding partners.

Inspection of the loadings obtained for this LDA analysis (see Figure S8 and Table S4 in the ESI) indicated that fluorescence emission measurements were the main contributors to both factors, with other measurements making minor contributions to the discrimination.

#### 3.3.3. Limit of Discrimination

We also determined the limit of discrimination for the [calcein•PAMAM] system by repeating the discrimination experiment at decreasing concentrations of analytes. At a 250 μM carboxylate concentration, i.e., ten times lower than the working concentration above, all carboxylates were still separated. Anion clusters first started to overlap at a 100 μM carboxylate concentration, losing resolution between fumarate and succinate. Accordingly, we estimated 100 μM < LOD < 250 μM, and in particular to lie close to 100 μM (see ESI, Figure S9). This is sufficient sensitivity for the envisioned applications: for instance, it would be acceptable for prostate cancer screening.

### 3.4. Unknown Quali-Quantitative Analysis

Standard curves for quantification. The excellent discrimination results shown above were obtained at a constant and known concentration of carboxylate analytes. Of course, however, in analytical practice the concentration of an unknown analyte is also unknown, so we needed to expand the method’s scope to the simultaneous determination of concentration and identity of each analyte. Our analysis of the origins of the discriminatory power of this sensing system had shown that fluorescence anisotropy, although an excellent report of binding, never was a strong contributor to the discrimination: it showed practically no differential response among carboxylates, i.e., it responded roughly equally to changes in concentration of any carboxylate anion in the analyte panel. This was exactly what we needed to detect only the concentration of any of the carboxylates of interest, independent of their identity. Once we determined their unknown concentration, we could dilute the sample appropriately and use the qualitative discrimination results we obtained above to determine their identity. Since fluorescence anisotropy measurements had already been routinely included in the original set of measurements, no change to our experimental protocols was required.

**Figure 10:**
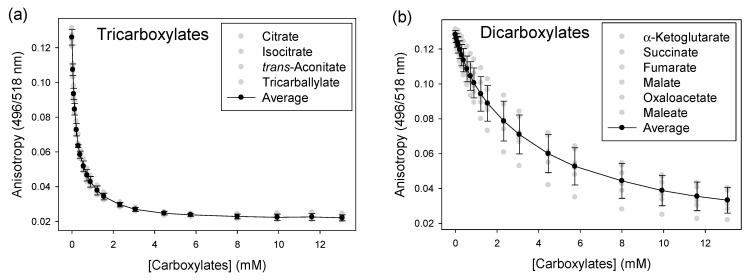
Fluorescence anisotropy standard curves for concentration determination for (**a**) tricarboxylates and (**b**) dicarboxylates. [calcein] = 6.36 μM, [G5] = 2.13 μM.

We also considered that tricarboxylates have a much higher affinity to PAMAM G5 than dicarboxylates, so we prepared two fluorescence anisotropy standard curves for concentration determination, as shown in Figure 10. Fluorescence anisotropy titrations were performed for all 10 carboxylate analytes. The titration profiles of the four tricarboxylates were averaged, generating a “tricarboxylate standard” curve for concentration determination. Similarly, a “dicarboxylate standard” curve was generated by averaging the titration profiles from the six dicarboxylates in our panel.

#### 3.4.1. Quantification of Unknown Samples

**Figure 11:**
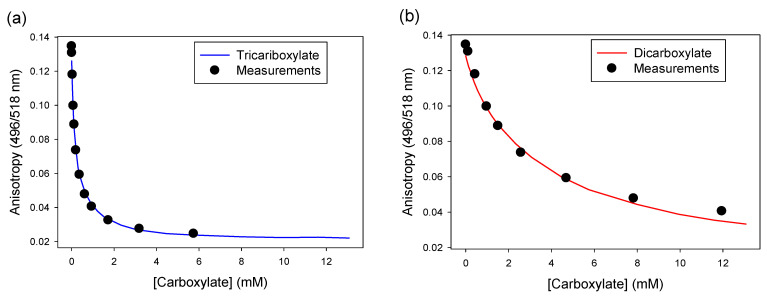
Concentration determination of unknown C, as a tricarboxylate (**a**) vs. as a dicarboxylate (**b**): line = dicarboxylate or tricarboxylate standard curve; dots = experimental values. Excitation: 496 nm, emission: 518 nm. [calcein] = 6.36 μM, [G5] = 2.13 μM.

Four unknown carboxylate solutions were used as titrants in a displacement titration with the [calcein•PAMAM] complex sensor, monitored by fluorescence anisotropy (Unknown **A** through **D**). Each titration profile (expressed as fluorescence anisotropy vs. volume of unknown added) was compared with the two standard curves mentioned above; the concentration of the unknown carboxylate in the sample was determined as that value which gave the best fit between the obtained titration profile and either one of the two standard curves. For example, in Figure 11 the anisotropy profile for Unknown **C** was fitted to both standard curves and its tentative concentration determined to be 34.4 mM as a tricarboxylate, or 436 mM as a dicarboxylate. Two new intermediate samples were then prepared by dilution of Unknown **C** to the 2.5 mM working concentration of our discrimination assay: a portion of Unknown **C** was diluted 13.76-fold and marked **C tri**; another was diluted 174.4-fold and marked **C di**. All four unknowns (**A**, **B**, **C**, **D**) were treated similarly; two intermediate samples were generated from each one (see ESI, Figure S11). These intermediate samples were then loaded on a 384-well microplate, together with true standards of the 10 carboxylates in our analyte panel at a 2.5-mM concentration.

#### 3.4.2. Identity and Concentration of Unknown Samples

We had previously ascertained that small variations in analyte concentration do not significantly change its cluster’s position in a PCA scores plot (Figure S10 in the ESI), so this technique is forgiving of small concentration errors arising from the preparation of intermediate samples by the dilution process described above.

**Figure 12:**
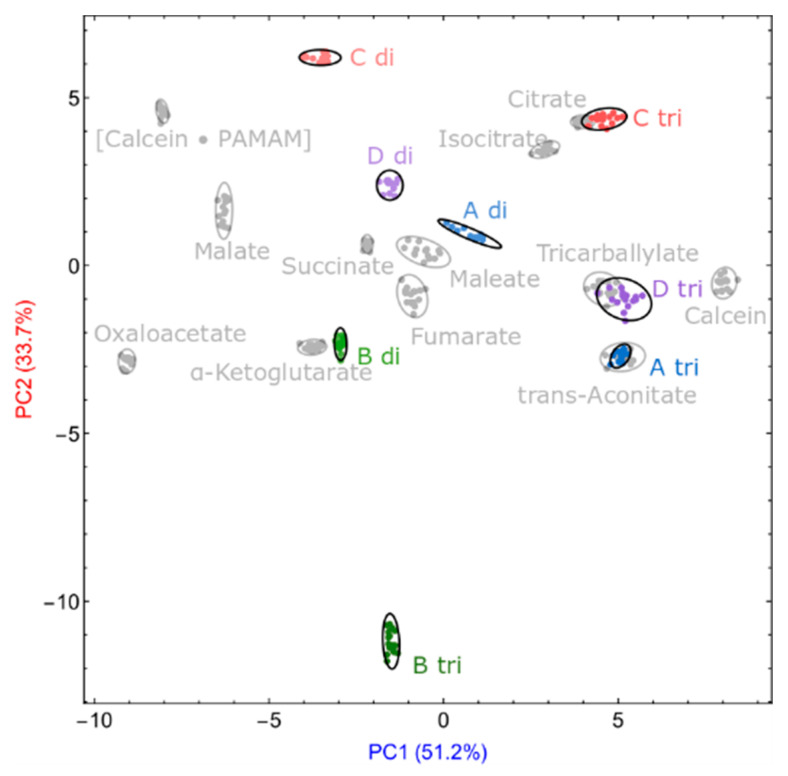
PCA scores plot for the simultaneous determination of identity and concentration of 4 unknown samples using the [calcein•PAMAM] complex sensor. In color: intermediate samples for each unknown; in gray: pure carboxylate reference samples. [calcein] = 6.36 μM, [PAMAM G5] = 2.13 μM, [reference carboxylates] = 2.50 mM. Performed in 50 mM aqueous HEPES buffer at pH 7.4, T = 25 °C.

Since the identity of each sample was unknown in this case, we selected the unsupervised PCA technique for data analysis and unknown identification. The resulting two-dimensional PCA scores plot is shown in Figure 12. Continuing to use Unknown **C** as an example, the “**C tri**” cluster was found to overlap with the citrate standard in this scores plot, while the “**C di**” sample did not overlap with any of the standards: thus, we concluded that Unknown **C** was a sample of citrate. Having found that the analyte in intermediate sample **C** was a tricarboxylate then allowed us to assign a concentration of 34.4 mM to the original Unknown **C**, which we had calculated for it on the hypothesis that it was a tricarboxylate.

Unknown **C** was in fact a sample of citrate at 38.5 mM, so the fingerprinting method described here correctly identified the identity of the carboxylate in the sample, as well as determined its concentration within a 10% error range.

The other samples were treated similarly. For instance, the **B tri** sample did not fall close to any of the standards, whereas the **B di** sample was found very close to α-ketoglutarate: thus, the identity of Unknown **B** was correctly assigned as α-ketoglutarate, and its concentration calculated as 23.1 mM (actual: 25.4 mM, 9% error). Unknowns **A** and **D** were analyzed similarly; all results are summarized in Table 1.

This was an excellent result for a synthetic pattern-based recognition system, considering that these systems are often altogether incapable of direct concentration determination.

## 4. Conclusions

A supramolecular sensing system built using non-covalent interaction from commercially available building blocks, an organic dye (calcein or pyranine), and a cationic polyelectrolyte (an amine-terminated PAMAM G5 dendrimer with ethylenediamine core) was used as a pattern-based sensor for biologically relevant carboxylates in water buffered to pH 7.4. Carboxylates were discriminated using absorbance, fluorescence emission, and fluorescence anisotropy measurements; the resulting dataset was analyzed with pattern recognition algorithms (principal component analysis and linear discriminant analysis) to extract chemical selectivity information from the nuanced interactions between the carboxylate analytes and the dye-polymer complexes. The [calcein•PAMAM] complex was the most effective, qualitatively differentiating all carboxylate analytes. The relationship between the analytes’ responses and their chemical structures was also elucidated.

This method was then extended to the simultaneous determination of the identity and concentration of carboxylates, with 100% accuracy for identification and 47% maximum error of quantification. Although a 47% error may appear large, this level of accuracy is acceptable for many of the applications envisioned here. In the screening for prostate cancer, for instance, diagnostically relevant anion levels can differ by multiple orders of magnitude, so even the worst of the results we presented would be viable in that context. The limit of discrimination for this method was found to be close to 100 μM.

Overall, this method provides simple and sensitive recognition and quantification of common carboxylates in notoriously challenging aqueous media using widely available high-throughput platforms.

## Figures and Tables

**Table 1:** Actual vs. experimentally determined concentrations of unknown samples.

Unk	Actual Identity	Concentration (mM)		% Error
		Calc’d	Actual	
A	*trans*-Aconitate	54.3	36.9	47.2
B	α-Ketoglutarate	23.1	25.4	9.06
C	Citrate	34.4	38.5	10.6
D	Tricarballylate	2.27	3.05	25.6

## Data Availability

Data generated in this study are available from the corresponding author.

## Supplementary Materials

The following are available online at https://www.mdpi.com/article/10.3390/s21113637/s1, Scheme S1: Organic dyes considered as candidate indicators. Table S1: p*K*_a_ of carboxylates and dyes, Table S2: Loadings for qualitative discrimination of carboxylates using pyranine, Table S3: Loadings for PCA analysis calcein, Table S4: Loadings for LDA analysis using calcein. Figure S1: Binding of calcein to PAMAM G5 dendrimer, Figure S2: Binding of α ketoglutarate to PAMAM G5, Figure S3: Binding of citrate, α ketoglutarate, fumarate, malate, and tricarballylate to PAMAM G5, Figure S4: outlier rejection, Figure S5: PCA scores plot for discrimination of carboxylates, Figure S6: LDA loadings plot for discrimination of carboxylates using pyranine. Figure S7: PCA loadings plot for discrimination of 10 carboxylates using calcein. Figure S8: LDA loadings plot for discrimination of carboxylates using calcein, Figure S9: LDA scores plots for limit of discrimination, Figure S10: PCA scores plot for small variation in concentration. Figure S11: Concentration analysis of unknown, Figure S12: PCA scores plot for simultaneous determination of identity and concentration.
