# Supplementary material for: Discrimination and Quantitation of Biologically Relevant Carboxylate Anions Using A [Dye•PAMAM] Complex
Source: Sensors (Basel). 2021 May 24;21(11):3637. doi: 10.3390/s21113637 (PMC8197244; doi:10.3390/s21113637)
Supplement: Supplementary file 1 [file sensors-21-03637-s001.zip › sensors-1225556-supplementary.pdf]

Supporting Information for:

Discrimination and quantitation of biologically relevant carboxylate anions using a [dye•PAMAM] complex

Yifei Xu,[a] Marco Bonizzoni*[a,b]

a. Department of Chemistry and Biochemistry, The University of Alabama, Tuscaloosa, AL, 35487 USA

b. Alabama Water Institute, The University of Alabama, Tuscaloosa, AL 35487 USA

Contents:

Experimental details S2
Instrumental parameters S4
Dye screening S6
pK_a of carboxylates and the dyes S7
Absorption and fluorescence spectra for the binding of calcein to PAMAM G5 .... S8
Absorption and fluorescence spectra for dye and anion binding with pyranine S9
Absorption and fluorescence spectra for dye and anion binding with calcein S10
Dataset workup before pattern recognition S12
PCA analysis for anion discrimination using the [pyranine•PAMAM] complex S13
LDA loadings for qualitative discrimination using [pyranine•PAMAM] S14
PCA loadings for qualitative discrimination using [calcein•PAMAM] S16
LDA loadings for qualitative discrimination using [calcein•PAMAM] S18
Limit of discrimination S20
Anion concentration has little effect on qualitative discrimination S21
Procedures for the determination of concentration and identity for unknown samples S22
References S25

Experimental details

Materials. Poly(amidoamine) (PAMAM) dendrimers were manufactured by Dendritech, Inc., and purchased as ~5 wt.% in methanol; the exact concentration of the lot used in this work was 1.419 mM. The final solutions used for all experiment contained negligible amount of methanol after dilution (< 0.8%). Dye solutions were prepared from calcein disodium salt, calcein blue, 5(6) -carboxyfluorescein, xylenol orange tetrasodium salt, glycine cresol red sodium salt, pyrogallol red, pyrocatechol violet, and naphthol yellow S purchased from Sigma Aldrich; pyranine, and naphthalene-1,3,6-trisulfonic acid trisodium salt hydrate from Alfa Aesar; alizarin red S from Acros. Carboxylate solutions were prepared from succinic acid, fumaric acid, *trans*-aconitic acid, DL-malic acid, α-ketoglutaric acid, and oxaloacetic acid purchased from Sigma Aldrich; maleic acid, and tricarballylic acid from Alfa Aesar; DL-isocitric acid trisodium salt hydrate from Acros; citric acid anhydrous from EMD Millipore; sodium acetate from Fisher Scientific. All solutions were made by dissolving solids or diluting stocks with DI water buffered with 50 mM 4-(2-hydroxyethyl) piperazine-1-ethanesulfonic acid (HEPES) purchased from IBI Scientific. Solution pH was adjusted to pH 7.4 by addition of solutions of NaOH and HCl, prepared from NaOH from Fisher Scientific and HCl from BDH Aristar. All materials were used as received.

Instrumentation. Benchtop absorbance titrations were carried out on a HP 8452A diode array spectrophotometer over the 230-800 nm spectral range, with a 2 nm resolution. An ISS PC1 spectrofluorimeter was used for fluorescence intensity and anisotropy titration measurements; the instrument is equipped with a broad-spectrum high-pressure xenon lamp (CERMAX, 300W) as the excitation light source, manual calibrated slits, and a rhodamine B quantum counter with a dedicated detector as the excitation correction. Motorized high-aperture Glan-Thompson calcite polarizers were used for fluorescence anisotropy measurement under computer control. A Hamamatsu red-sensitive PMT operating in photon-counting mode was used as detector. An external circulating water bath was used to maintain the cuvette temperature as 25 °C for all titration experiments. Benchtop experiments were performed in 1 cm square Starna fused quartz cuvettes.

A Biotek Synergy II multimode plate reader was used for the microwell plate-based discrimination studies. The light source was a tungsten lamp providing a continuum spectrum in the visible range. Absorbance spectra were measured through a monochromator; fluorescence intensity was measured through bandpass filters; and fluorescence anisotropy was measured through plastic sheet polarizers and bandpass filters. For fluorescence measurements were conducted in "top-detected" mode: both the excitation and collection of emitted light took place from the top side of the plate; a dichroic mirror was placed between sample and emission channel to block the excitation light from reaching the detector. The temperature in the sample compartment was controlled with an electric heater integrated in the instrument.

Greiner BioOne non-treated polystyrene 384-well plate with black walls and clear bottoms were used. Sample solutions were deposited on the plates by hand using Eppendorf Research multichannel pipettors with disposable plastic tips. Total solution volume in each well was kept constant at 100 µL exactly. Plates were read immediately after sampling and never reused.

Details on the wavelength selection and conditions for microwell measurements are summarized in the following Instrumental Parameters section.

Titration Conditions. Concentrations for dye binding titrations were: [calcein] = 6.36 µM or [pyranine] = 6.04 µM; and concentrations for anion binding titrations were: [calcein] = 6.36 µM + [G5] = 2.13 µM, or [pyranine] = 6.04 µM + [G5] = 0.213 µM.

General Dye Binding Titration Protocol. Binding of the dye to PAMAM G5 was studied by titrating a "titrant" solution which contained dye and PAMAM G5, into a "cuvette" solution which contained the dye at the same concentration, so no change in concentration of the chromophore would take place during the course of the titration. Benchtop titrations were performed by addition of aliquots of titrant to 2 mL of the cuvette solution in a 1 cm quartz cuvette.

General Anion Binding Titration Protocol. In this case the "cuvette" solution for displacement contained the relevant [dye•PAMAM] complex with the concentration stated above, and the "titrant" solution contained [dye•PAMAM] complex with the same concentration, as well as a carboxylate at a concentration about 5000 times higher than the dye. Benchtop titrations for displacement were carried out in the same way as dye binding: the concentration of the [dye•PAMAM] sensing complex was kept constant, while the concentration of carboxylate analytes was increased.

Multiwell Plate Experiments. Absorbance, fluorescence intensity, and fluorescence anisotropy were measured sequentially on the same plate to ensure consistency. Aqueous HEPES buffer (50 mM at pH 7.4) was used as blank. Each carboxylate sample was deposited in 36 replicates for discrimination experiment, and 16 replicates for the unknown identity and concentration determination experiments. Data analysis was carried out in Wolfram Mathematica using Principal Component Analysis (PCA) and Linear Discriminant Analysis (LDA) algorithms implemented in-house.

Simultaneous determination of unknown identity and concentration. Analytes were separated into two groups according to their charge status, i.e. dicarboxylates and tricarboxylates. Two separate standard curves were prepared by averaging the fluorescence anisotropy displacement profiles, one for each group of analytes. Unknown samples were prepared by our co-worker Dr. Xiaoli Liang. Unknowns were adjusted to pH 7.4 and displacement titrations were run as previously described. The resulting fluorescence anisotropy profiles for each unknown were superimposed onto both standard curves, to estimate the concentration of the unknown, in each case under the hypothesis of the unknown being a tricarboxylate vs. a dicarboxylate, resulting in two estimated concentrations for each unknown sample.

Two intermediate samples were then generated from each unknown, by dilution to 2.5 mM based on the two estimated concentrations. These intermediate samples were then deposited on a 384-well plate, together with pure carboxylate standards at 2.5 mM.

Data analysis was then carried out using PCA to obtain scores plots. Discrimination was performed by comparing the distance between the unknown clusters and the reference clusters. In all cases, *only one* of the clusters from the two intermediate samples generated for each unknown matched with one of the reference clusters. This allowed us to determine the identity of the unknown, which then automatically validated one of the two tentative concentrations we had calculated for each unknown sample.

Instrumental parameters

Pyranine

Benchtop:

• Excitation wavelength:	418 nm (dye binding), 420 nm (dye displacement)
• Emission spectra:	450-580 nm
• Slit, excitation ch.:	4 nm spectral resolution
• Slit, emission ch.:	4 nm spectral resolution
• Filter, emission ch.:	0.3 AU neutral density
• Iris:	open
• Lamp current:	18 A
• Sample temperature:	25 °C

Plate reader:

• Absorbance:	300, 310, 320, 330, 340, 350, 360, 370, 380, 390, 400, 405, 425, 430, 435, 440, 445, 450, 460, 465, 470 nm
• Excitation filters:	380 ± 20 nm, 450 ± 50 nm, 460 ± 40 nm, 485 ± 20 nm
• Emission filters:	516 ± 20 nm, 528 ± 20 nm, 560 ± 40 nm, 580 ± 50 nm
• Polarizers:	plastic film, only for anisotropy measurements
• Detector gain:	automatically adjusted by reading the whole plate and setting the highest reading to 80% of the available detector range.

Calcein

Benchtop:

- Excitation wavelength: the choice was based on the position of the isosbestic point
  496 nm (citrate, malate)
  486 nm (fumarate, α-ketoglutarate, and tricarballylate)
- Emission range: 500-600 nm
- Emission λ for anisotropy: 518 nm
- Slit, excitation: 2 nm spectral resolution for emission,
  8 nm spectral resolution for anisotropy
- Slit, emission: 2 nm spectral resolution for all conditions
- Iris: open
- Lamp current: 18 A
- Sample temperature: 25 °C

Plate reader:

- Absorbance: 300, 310, 320, 330, 340, 350, 360, 440, 450, 460, 470, 480, 486, 492, 494, 496, 500, 510, 520 nm
- Excitation filter: 460 ± 40 nm, 485 ± 20 nm
- Emission filter: 516 ± 20 nm, 528 ± 20 nm, 560 ± 40 nm
- Dichroic mirror: 510 nm cutoff for anisotropy measurements
- Polarizers: plastic film, only for anisotropy measurements
- Detector gain: automatically adjusted by reading the whole plate and setting the highest reading to 80% of the available detector range.

Dye screening

The dyes shown in Scheme S1 were considered to build a [dye•PAMAM] sensing complex, but they were ultimately eliminated from consideration. For example, although naphthalene-1,3,6-trisulfonate binds to PAMAM G5 and can be displaced by carboxylate analytes, nevertheless its absorption lies in the UV range, which is incompatible with the polystyrene material used in the plate-based studies. Alizarin red S binds to PAMAM G5 at pH 7.4, however the change in spectroscopic properties upon binding is so small that it would have been ineffective for discrimination. On the other hand, glycine cresol red binds strongly to PAMAM G5 and provides a good dynamic range, but it was relatively indiscriminate in its binding response, contributing no discriminatory power among carboxylates. Similar reasons applied to the other dyes considered here, which were studied no further.

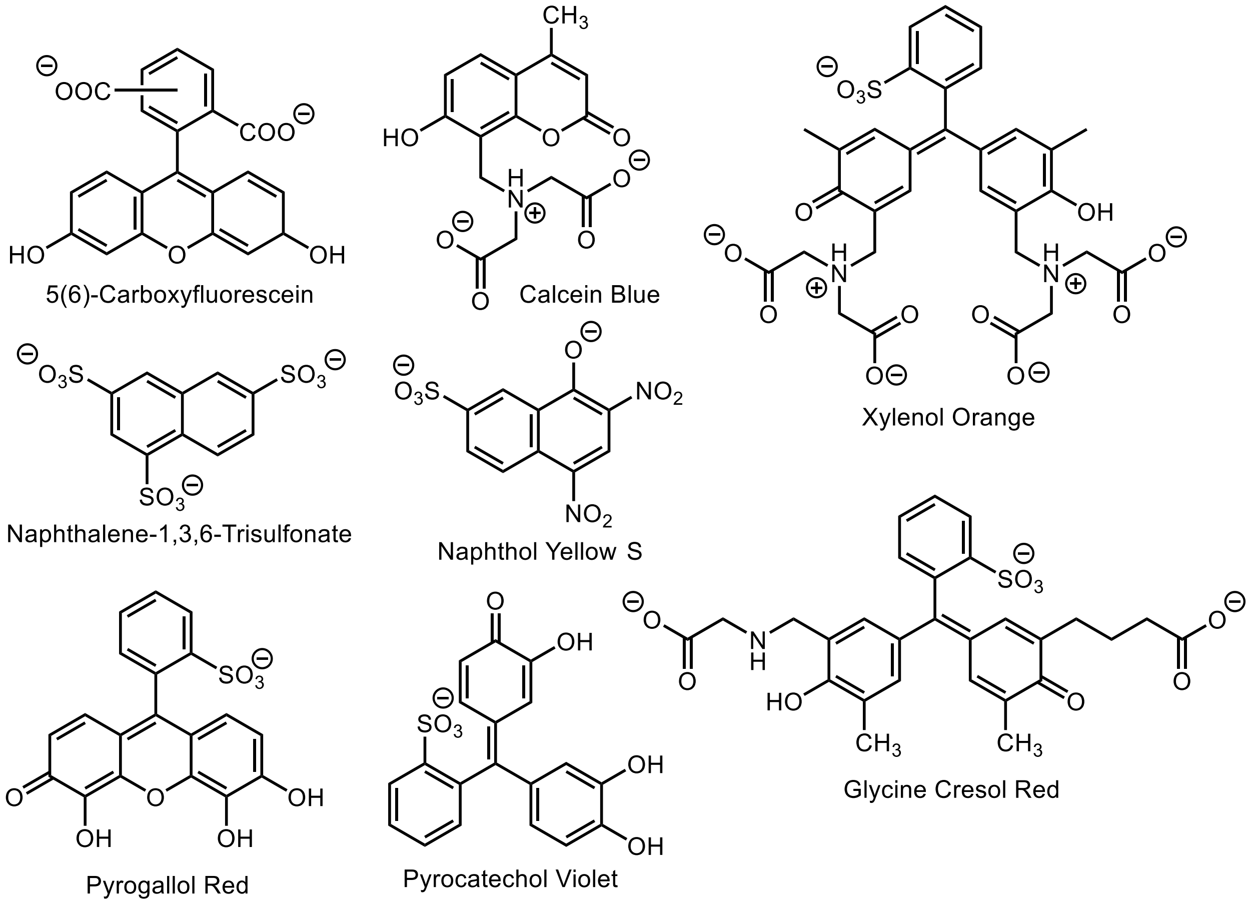

Scheme S1. Organic dyes that were considered and rejected as candidate indicators in the construction of a supramolecular [dye•PAMAM G5] sensing complex.

pK_a of carboxylates and dyes

Table S1. pK_a of carboxylates and dyes.

**Name**	**pK_{a1}**	**pK_{a2}**	**pK_{a3}**	**pK_{a4}**	**pK_{a5}**	**pK_{a6}**
Citrate[1]	3.13	4.76	6.40			
Isocitrate[1]	3.29	4.71	6.40			
Tricarballylate[2]	3.49	4.58	5.83			
trans-Aconitate[3]	2.91	4.33	6.16			
α-Ketoglutarate[4]	2.35	4.85				
Succinate[1]	4.21	5.64				
Fumarate[1]	3.02	4.38				
Malate[1]	3.40	5.11				
Oxaloacetate[1]	2.55	4.37				
Maleate[1]	1.92	6.23				
Pyranine[5]	7.2					
Calcein[6]	2.1	2.9	4.2	5.5	10.8	11.7

Absorption and fluorescence spectra for the binding of calcein to PAMAM G5

The family of spectra associated with the binding of calcein to PAMAM G5 are shown in Figure S1 below; the corresponding profile is included in Figure 4 in the main manuscript. Small aliquots of a concentrated solution of PAMAM G5 and 6.36 μM of calcein in 50 mM aqueous HEPES buffer at pH 7.4 were added to a 1 cm quartz cuvette containing 2 mL of 6.36 μM of calcein solution. The titrant solution contained the dye at the same concentration as the cuvette solution to avoid dilution effects. The absorbance peak characteristic of the free dye shifted about 6 nm towards the red, while showing a small increase in intensity. The fluorescence emission decreased sharply, then increased and reached a plateau, as discussed in the manuscript.

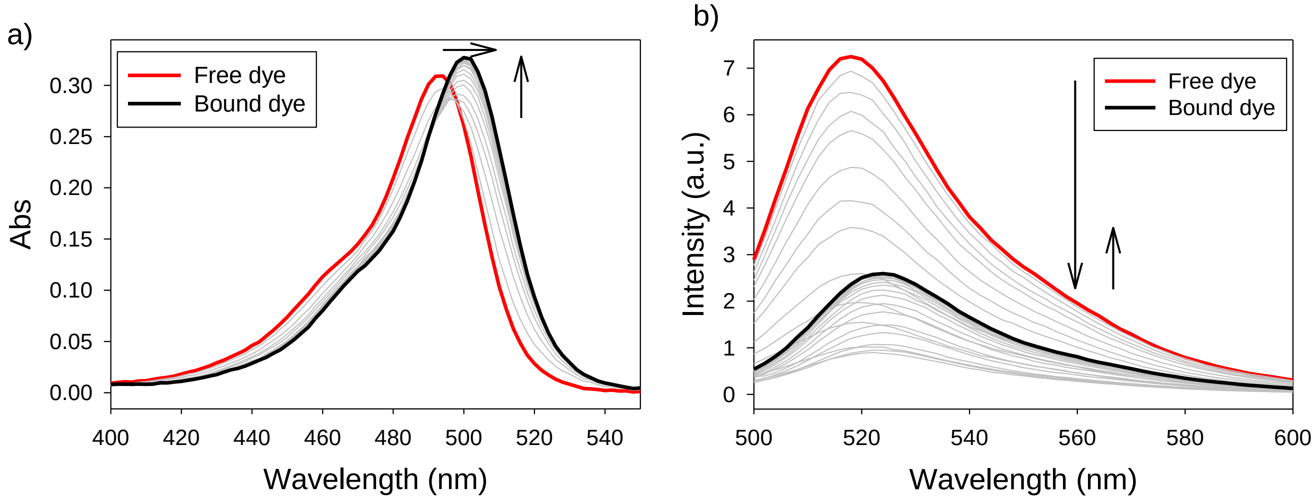

Figure S1. Binding of calcein to PAMAM G5 dendrimer. a) Absorbance spectra; b) Fluorescence emission spectra. The titration was performed in 50 mM aqueous HEPES buffer at pH 7.4, T = 25 °C. [calcein] = 6.36 μM, excitation: 496 nm.

Absorption and fluorescence spectra for dye and anion binding with pyranine

The displacement titration spectra are shown in Figure S2 below (the corresponding profile is included in Figure 5 in the main manuscript). Aliquots of a concentrated α-ketoglutarate solution (also containing [pyranine] = 6.04 μM, [PAMAM G5] = 0.213 μM) were added to a cuvette containing 2 mL of the [pyranine•PAMAM] complex ([pyranine] = 6.04 μM, [PAMAM G5] = 0.213 μM). The titrant solution contained dye and PAMAM dendrimer at the same concentration as the cuvette solution, to avoid dilution effects. During the titration both the absorbance and the fluorescence emission signal went back to the one associated with the free dye in solution, indicating that the dye was displaced from its complex and, indirectly, that α-ketoglutarate was able to bind to the dendrimer. The increase in absorbance around 300 nm is the absorbance of α-ketoglutarate.

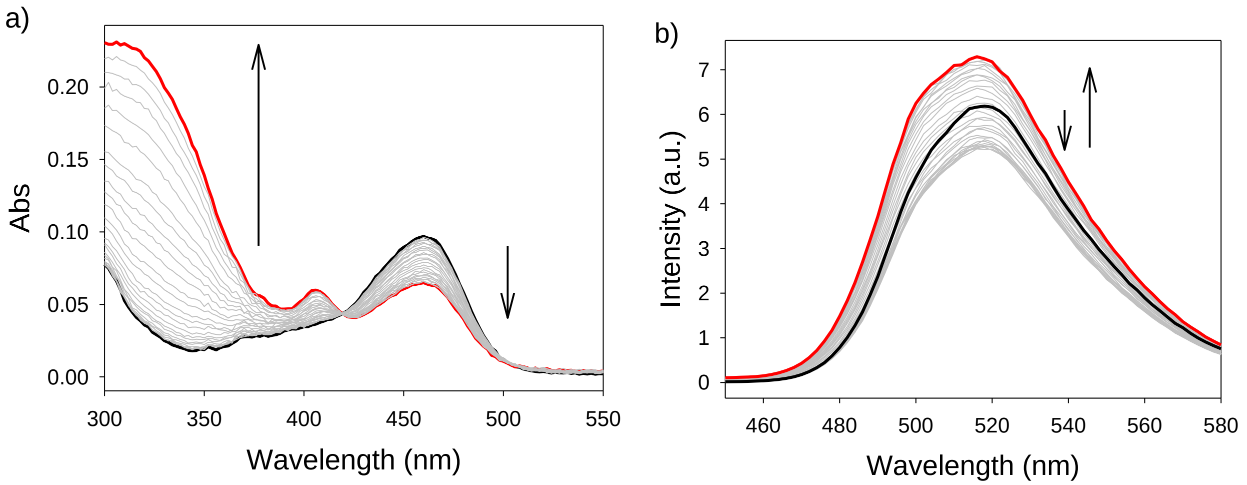

Figure S2. The binding of α-ketoglutarate to PAMAM G5 is indicated by the displacement of the pyranine dye from its complex with PAMAM G5 as aliquots of α-ketoglutarate are added to the solution. a) Absorbance spectra; b) Fluorescence emission spectra. The spectrum in black was the first titration point; the one in red was the last. Performed in 50 mM aqueous HEPES buffer at pH 7.4, T = 25 °C. [pyranine] = 6.04 μM, [PAMAM G5] = 0.213 μM.

Absorption and fluorescence spectra for dye and anion binding with calcein

The displacement titration spectra are shown in Figure S3 below (the corresponding profile is included in Figure 6 in the main manuscript). Aliquots of a concentrated carboxylate solution (also containing [calcein] = 6.36 μM, [PAMAM G5] = 2.13 μM) were added to a cuvette containing 2 mL of the [calcein•PAMAM] complex ([calcein] = 6.36 μM, [PAMAM G5] = 2.13 μM). The titrant solution contained the dye and PAMAM at the same concentration as the cuvette solution to avoid dilution effects. During the titration both the absorbance and the fluorescence emission signal went back to the one associated with the free dye in solution, indicating that the dye was displaced from its complex and, indirectly, that carboxylates were able to bind to the dendrimer.

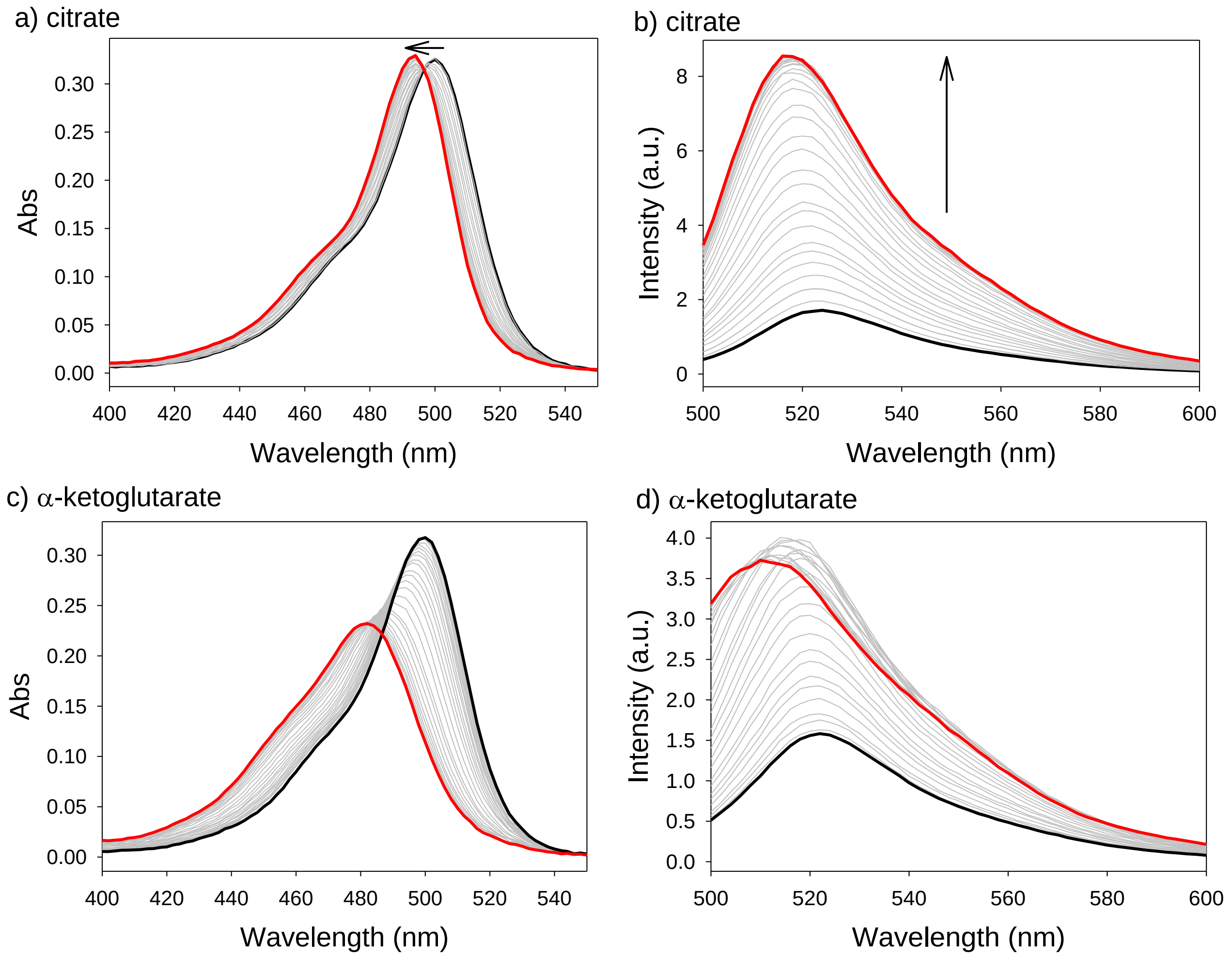

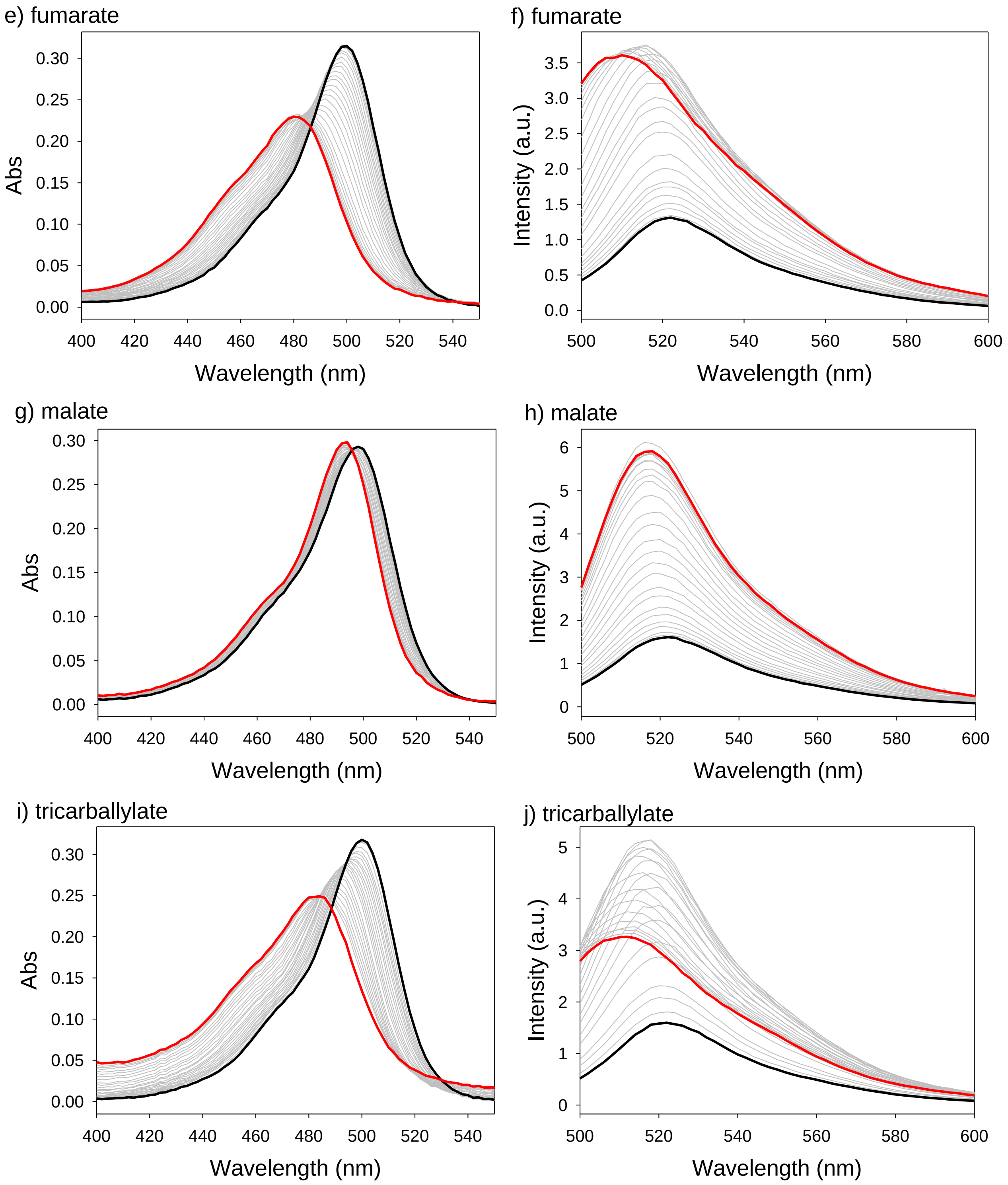

Figure S3. The binding of citrate, α-ketoglutarate, fumarate, malate, and tricarballylate to PAMAM G5 is indicated by the displacement of the calcein dye from its complex with PAMAM G5 as aliquots of anion are added to the solution. left: Absorbance spectra; right: Fluorescence emission spectra. The spectrum in black was the first titration point; the one in red was the last. Performed in 50 mM aqueous HEPES buffer at pH 7.4, T = 25 °C. [calcein] = 6.36 μM, [PAMAM G5] = 2.13 μM.

Dataset workup before pattern recognition

Data sets were worked up in several steps before final pattern recognition using PCA or LDA analysis. First, the averaged value of the blank readings was automatically subtracted from the spectroscopic data acquired on the plate reader. Then, the variables that showed no response (too noisy) or that showed similar responses toward all analytes were removed by hand, since they would not have contributed to the discriminatory power of the system but would have introduced undesirable noise.

Outlier tests were then carried out for each group of replicates corresponding to a single sample. Figure S4 shows an example of outlier tests for the *trans*-aconitate set of replicates obtained from the qualitative carboxylate differentiation plate using the [calcein•PAMAM] complex sensor (see Figure 9 in the main manuscript). The raw measurements for the 36 replicates of *trans*-aconitate were fed to Principal Component Analysis (PCA), then a 2D PCA scores plot was generated using the first two principal component as coordinates. A 95% bivariate confidence ellipsoid was also calculated. As shown below, replicate #7, in red, fell outside the 95% confidence ellipsoid so it was removed from the dataset. Typical causes we observed for such outliers may be mistakes in sample deposition (when using handheld multichannel pipettes for small volumes), imperfections in the plate's wells, or scratches on the clear bottom of the plate.

After performing raw measurement selection and outlier rejection, the remaining data was subjected to PCA and LDA analysis.

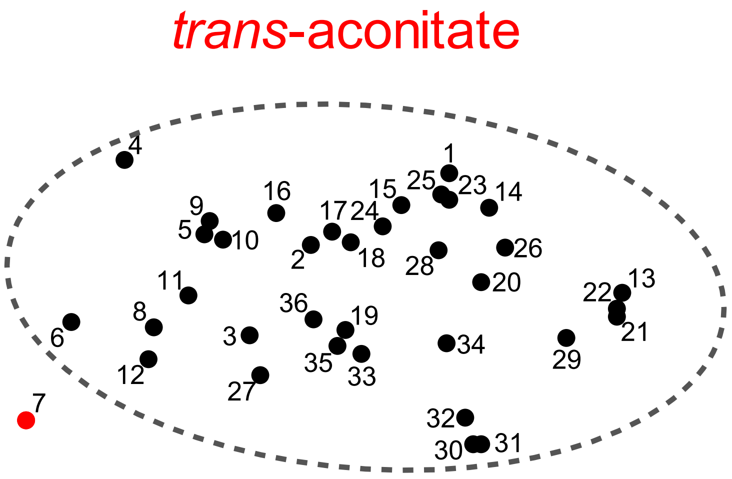

Figure S4. The data on which we based outlier rejection from the 384-well plate result for qualitative carboxylate recognition, using *trans*-aconitate as an example. Performed on a 384-well plate in 50 mM HEPES buffer at pH 7.4, T = 25 °C. [calcein] = 6.36 μM, [G5] = 2.13 μM, [*trans*-aconitate] = 2.30 mM.

PCA analysis for anion discrimination using the [pyranine•PAMAM] complex

The results of Principal Component Analysis (PCA) for qualitative differentiation of carboxylates using the [pyranine•PAMAM] complex are shown in Figure S5 below as a 2D PCA scores plot. This result was obtained from the same dataset used for LDA discrimination, whose results are shown in Figure 7 in the main manuscript.

Unfortunately, discrimination using PCA was incomplete for this sensor. Nevertheless, we could still distinguish two large groupings of carboxylates, a tricarboxylate supercluster (yellow), closer to the pyranine reference, and a dicarboxylate supercluster (green), closer to the [pyranine•PAMAM] "bound dye" reference (oxaloacetate alone does not conform to this behavior, probably due to its uniquely high intrinsic UV absorbance). These groupings are consistent with the superclusters identified in the results of LDA analysis and derive from the same structural effects.

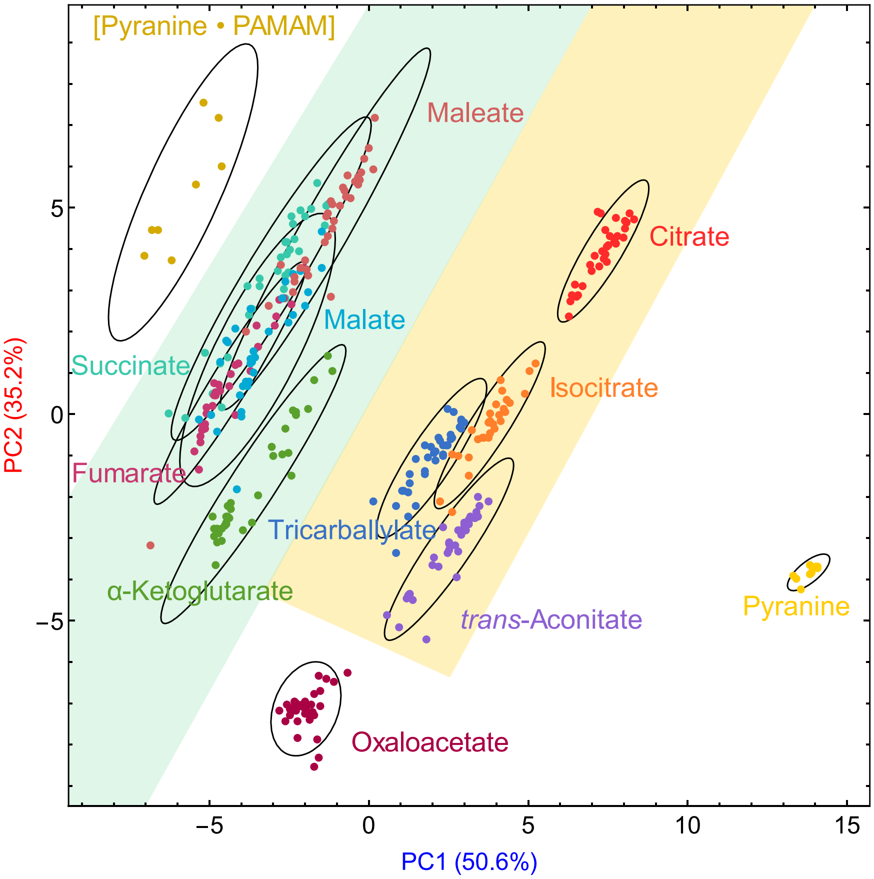

Figure S5. PCA 2D scores plot for the discrimination of the 10 carboxylates in our analyte pool using [pyranine•PAMAM G5]. Percentages in each axis titles indicate the fraction of information from the original dataset that is represented by that axis. This scores plot thus reproduces a total of 85.8% of the information contained in the original dataset. The experiment was performed on a 384-well plate in 50 mM aqueous HEPES buffer at pH 7.4, T = 25 °C. [pyranine] = 6.04 µM, [G5] = 0.213 µM, [carboxylates] = 2.04 mM.

LDA loadings for the qualitative discrimination of carboxylates using the [pyranine•PAMAM] complex sensor

A loadings plot is a graphic representation of the contribution of each raw instrumental measurement (e.g. absorbance at a certain wavelength, fluorescence emission at a certain wavelength combination) to the discrimination of analytes along each factor. Two-dimensional loadings plots are the most common, for their ease of presentation. Below is the LDA loadings plot obtained using the [pyranine•PAMAM] complex as sensor (the corresponding LDA scores plot is in Figure 7 in the main manuscript). The loadings often allow a better understanding of the underlying physical properties driving the separation observed in the corresponding scores plot.

In this case, the differentiation along factor 1 is due to absorbance measurements for the most part, with a particularly high contribution from absorbance at 330 nm. On the other hand, differentiation along factor 2 is mostly due to fluorescence emission measurements. The same information is presented in a tabular form in Table S2, including contributions to factor 3 as well.

Perusal of the contributions to factor 3 also indicates that the contributing measurements in factor 3 have significant similarities to factor 2: thus, a three-dimensional scores plot would have produced only marginally better differentiation, while being much harder to present.

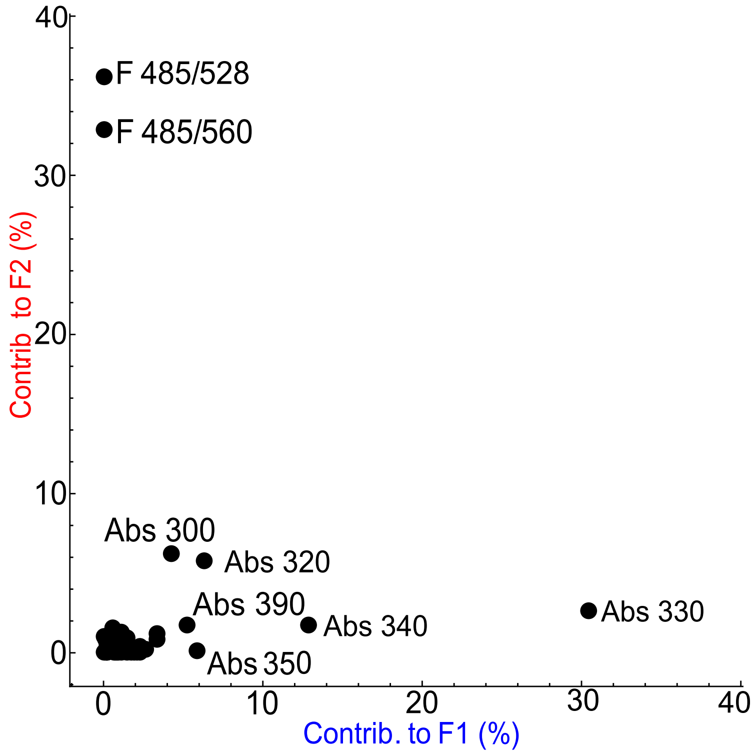

Figure S6. LDA loadings plot for the first two factors obtained in the discrimination of the 10 carboxylates in our analyte pool using [pyranine•PAMAM G5]. Each point represents the contribution of the corresponding measurement and is labeled with the relevant wavelength(s); Abs = absorbance at a specified wavelength; F = fluorescence intensity with the corresponding $\lambda_{ex}/\lambda_{em}$ (in nm). Instrumental measurements contributing less than ca. 5% to either factor were included but not labeled, for clarity. The experiment was performed on a 384-well plate in 50 mM aqueous HEPES buffer at pH 7.4, T = 25 °C. [pyranine] = 6.04 μM, [G5] = 0.213 μM, [carboxylates] = 2.04 mM.

Table S2. Loadings for all measured variables for the qualitative discrimination of carboxylates using pyranine-G5 complex as sensor. F*n*% (columns) is the percent of the information explained by Factor *n* that is provided by each raw measurement (rows).

Variable type	Wavelength (nm)	F1%	F2%	F3%
Absorbance	300	4	6	20
Absorbance	310	1	0	10
Absorbance	320	6	6	1
Absorbance	330	30	3	0
Absorbance	340	15	2	1
Absorbance	350	6	0	0
Absorbance	360	0	0	0
Absorbance	370	1	1	1
Absorbance	380	3	1	0
Absorbance	390	5	2	0
Absorbance	400	3	1	0
Absorbance	405	2	0	0
Absorbance	425	1	0	0
Absorbance	430	1	0	0
Absorbance	435	1	0	0
Absorbance	440	2	0	0
Absorbance	445	0	0	0
Absorbance	450	1	2	0
Absorbance	455	0	0	0
Absorbance	460	2	0	0
Absorbance	465	1	0	0
Absorbance	470	1	1	0
Fluorescence emission	380/516	3	0	0
Fluorescence emission	380/528	2	0	1
Fluorescence emission	380/560	0	1	4
Fluorescence emission	380/580	0	1	3
Fluorescence emission	450/516	1	0	0
Fluorescence emission	450/528	1	0	0
Fluorescence emission	450/560	0	0	0
Fluorescence emission	450/580	1	0	0
Fluorescence emission	460/516	0	1	0
Fluorescence emission	460/528	2	1	1
Fluorescence emission	460/560	2	0	0
Fluorescence emission	460/580	0	0	1
Fluorescence emission	460/516	1	1	1
Fluorescence emission	460/528	0	36	36
Fluorescence emission	460/560	0	33	19
Fluorescence emission	460/580	0	1	0

PCA loadings for the qualitative discrimination of carboxylates using the [calcein•PAMAM] complex sensor

The following plot presents the loadings (i.e. contributions of each raw measurement to the principal components) obtained by PCA analysis for the qualitative differentiation of carboxylates using the [calcein•PAMAM] sensor (the corresponding PCA scores plot is in Figure 8 in the main manuscript). Unlike LDA, in which some measurements had low contribution to both factors (see next section), all raw measurements contribute significantly here. Absorbance, fluorescence emission, and fluorescence anisotropy all contribute to the first principal component (PC1), while discrimination along the second component (PC2) is mostly due to differences in absorbance at a different set of wavelengths. The same data is tabulated in Table S3.

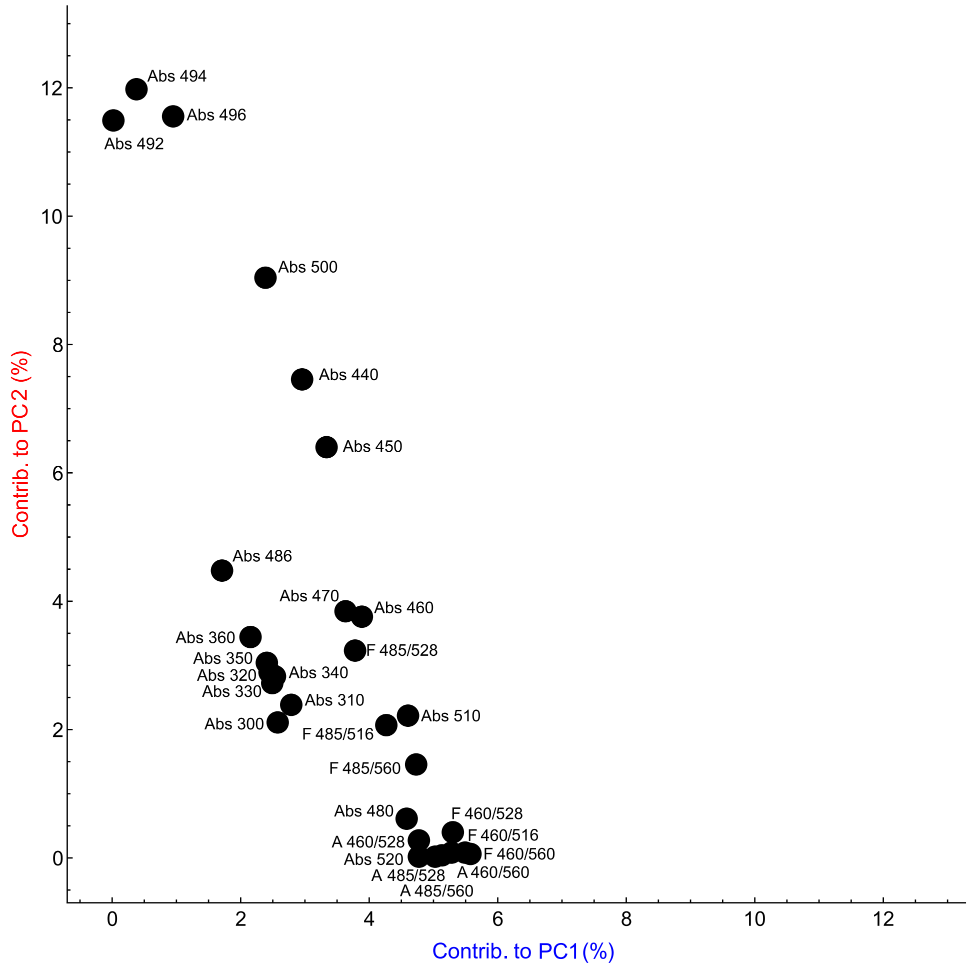

Figure S7. PCA loadings plot for the discrimination of the 10 carboxylates in our analyte pool using [calcein•PAMAM G5]. Each point represents the contribution of the corresponding measurement and is labeled with the relevant wavelength(s); Abs = absorbance at a specified wavelength; F = fluorescence intensity, A = fluorescence anisotropy with the corresponding $\lambda_{ex}/\lambda_{em}$ (in nm). The experiment was performed on a 384-well plate in 50 mM aqueous HEPES buffer at pH 7.4, T = 25 °C. [calcein] = 6.36 µM, [G5] = 2.13 µM, [carboxylates] = 2.30 mM.

Table S3. Loadings for all raw measurements from PCA analysis for the differentiation of carboxylates using the [calcein•PAMAM G5] complex. PC*n*% (column) is the percentage of the information carried by principal component *n* that was originally present in the corresponding raw measurement. (row).

**Variable type**	**Wavelength (nm)**	**PC1%**	**PC2%**
Absorbance	300	3	2
Absorbance	310	3	2
Absorbance	320	2	3
Absorbance	330	2	3
Absorbance	340	3	3
Absorbance	350	2	3
Absorbance	360	2	3
Absorbance	440	3	7
Absorbance	450	3	6
Absorbance	460	4	4
Absorbance	470	4	4
Absorbance	480	5	1
Absorbance	486	2	4
Absorbance	492	0	11
Absorbance	494	0	12
Absorbance	496	1	12
Absorbance	500	2	9
Absorbance	510	5	2
Absorbance	520	5	0
Fluorescence emission	460/516	6	0
Fluorescence emission	460/528	5	0
Fluorescence emission	460/560	5	0
Fluorescence emission	485/516	4	2
Fluorescence emission	485/528	4	3
Fluorescence emission	485/560	5	1
Fluorescence anisotropy	460/528	5	0
Fluorescence anisotropy	460/560	5	0
Fluorescence anisotropy	485/528	5	0
Fluorescence anisotropy	485/560	5	0

LDA loadings for the qualitative discrimination of carboxylates using the [calcein•PAMAM] complex sensor

These are the corresponding loadings (i.e. contributions of each raw measurement to the factors) obtained from LDA analysis for the qualitative differentiation of carboxylates using the same system as above, i.e. [calcein•PAMAM] sensor. The same data is tabulated in Table S4.

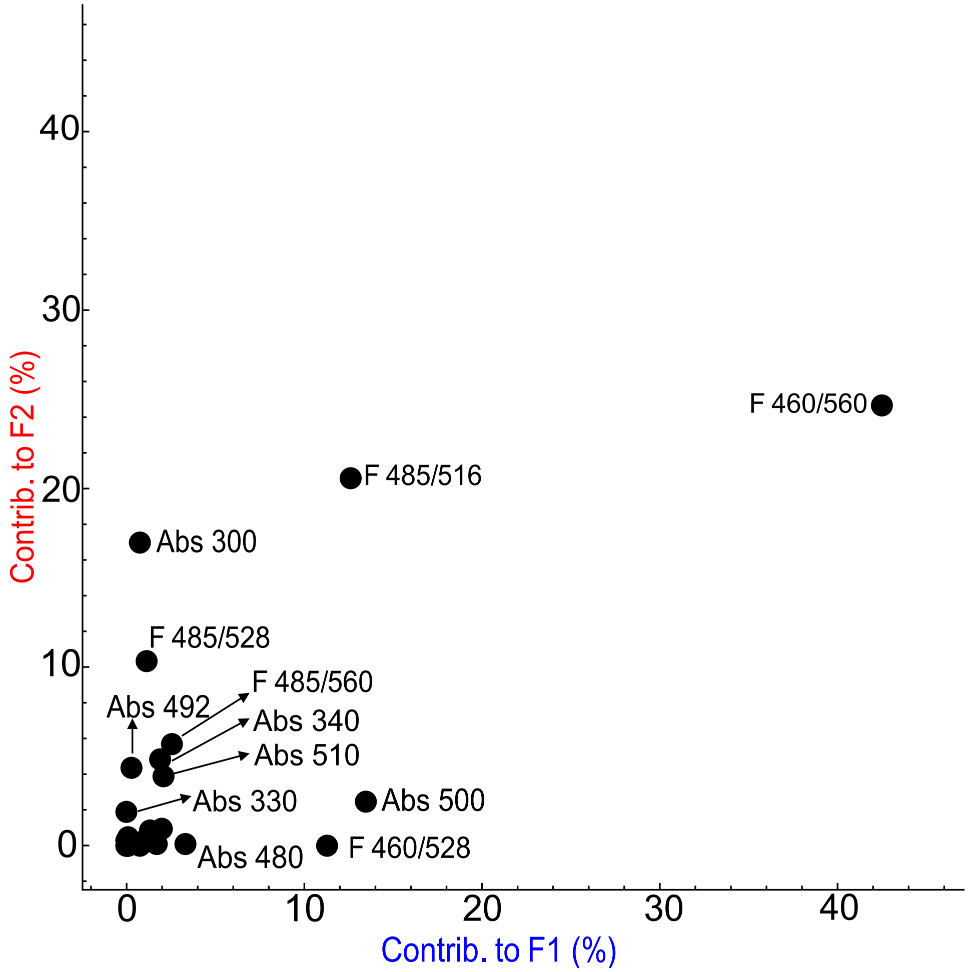

Figure S8. LDA loadings plot for the discrimination of the 10 carboxylates in our analyte pool using the [calcein•PAMAM G5] complex. Each point represents the contribution of the corresponding measurement and is labeled with the relevant wavelength(s); Abs = absorbance at a specified wavelength; F = fluorescence intensity with the corresponding $\lambda_{ex}/\lambda_{em}$ (in nm). Instrumental measurements contributing less than ca. 5% to either factor were included but not labeled, for clarity. The experiment was performed on a 384-well plate in 50 mM aqueous HEPES buffer at pH 7.4, T = 25 °C. [calcein] = 6.36 µM, [G5] = 2.13 µM, [carboxylates] = 2.30 mM.

Table S4. Loadings for all raw measurements from LDA analysis for the qualitative discrimination of carboxylates using [calcein•PAMAM G5] complex. F*n*% (columns) represent the percent of the information explained by Factor *n* that is provided by each raw measurement (rows).

Variable type	Wavelength (nm)	F1%	F2%	F3%
Absorbance	300	1	17	32
Absorbance	310	1	1	0
Absorbance	320	2	0	1
Absorbance	330	0	2	7
Absorbance	340	0	5	5
Absorbance	350	0	0	0
Absorbance	360	0	0	4
Absorbance	440	0	0	1
Absorbance	450	0	0	0
Absorbance	460	1	0	0
Absorbance	470	1	0	0
Absorbance	480	3	0	0
Absorbance	486	0	0	0
Absorbance	492	0	4	1
Absorbance	494	1	0	0
Absorbance	496	2	1	0
Absorbance	500	13	2	0
Absorbance	510	2	4	3
Absorbance	520	0	0	0
Fluorescence emission	460/516	0	0	8
Fluorescence emission	460/528	11	0	0
Fluorescence emission	460/560	42	25	15
Fluorescence emission	485/516	13	21	7
Fluorescence emission	485/528	1	10	5
Fluorescence emission	485/560	3	6	9
Fluorescence anisotropy	460/528	0	0	0
Fluorescence anisotropy	460/560	0	0	0
Fluorescence anisotropy	485/528	0	0	0
Fluorescence anisotropy	485/560	0	0	0

Determination of the limit of discrimination

The limit of discrimination was estimated by repeating the qualitative discrimination experiment using the [calcein•PAMAM] sensor, at decreasing concentrations of carboxylate analytes. Figure S9 shows that at a concentration of 250 µM (Figure S9a) the carboxylates were still well separated, whereas at a concentration of 100 µM (Figure S9b) the fumarate and succinate clusters started to overlap. The limit of discrimination of our system could therefore be estimated to be higher than but close to 100 µM.

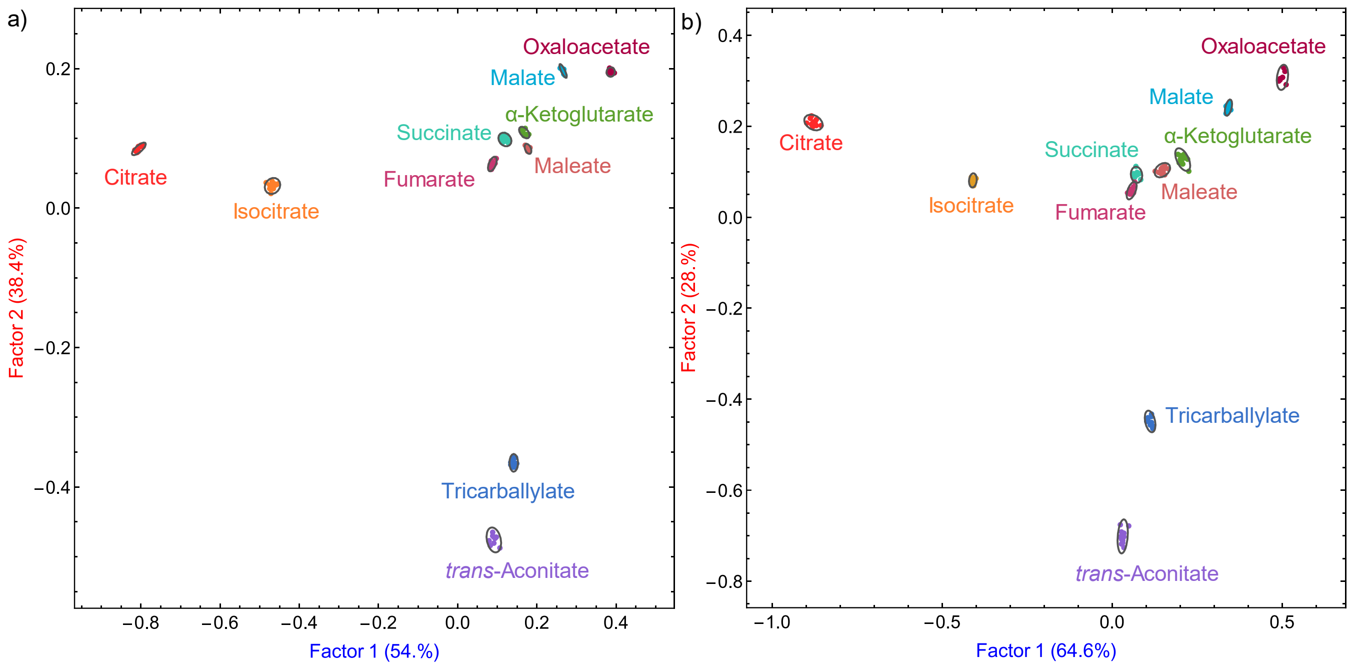

Figure S9. LDA 2D scores plots for the discrimination of 10 carboxylates using [calcein•PAMAM] at decreasing concentration of carboxylate, for the determination of the limit of discrimination. Percentages in the axis title indicate the fraction of information from the original dataset that is summarized by each factor. a) [carboxylates] = 250 µM; b) [carboxylates] = 100 µM. Performed on a 384-well plate in 50 mM aqueous HEPES buffer at pH 7.4, T = 25 °C. For clarity, the free calcein and [calcein•PAMAM] reference samples are not shown here, to focus on the carboxylate samples.

Anion concentration has little effect on qualitative discrimination

To ascertain whether a small change in concentration might severely affect the qualitative determination of the identity of carboxylate anions, a qualitative discrimination experiment was conducted with 10 carboxylates at a 2.5 mM concentration (as a reference), as well as with 5 of these carboxylates (oxaloacetate, succinate, isocitrate, fumarate, tricarballylate) at a slightly lower (2.0 mM) and slightly higher (3.0 mM) concentration.

Figure S10 shows that clusters of the same carboxylate at different concentrations were always found very close to each other, if not overlapping. The relative positions of these clusters also reflected the extent of carboxylate binding: at different concentrations, the lowest-concentration cluster (2.0 mM) was always found closest to the sensing complex reference (i.e. the fully bound dye), whereas the highest-concentration one (3.0 mM) was closest to the free dye reference. This is consistent with the fact that, with less anion, the extent of carboxylate binding was lower, so less dye was being displaced.

Overall, we could conclude that the qualitative separation of different carboxylates was dominated by their structure and it was tolerant of small changes in concentration that may be inadvertently caused by sample preparation and dilution before measurement.

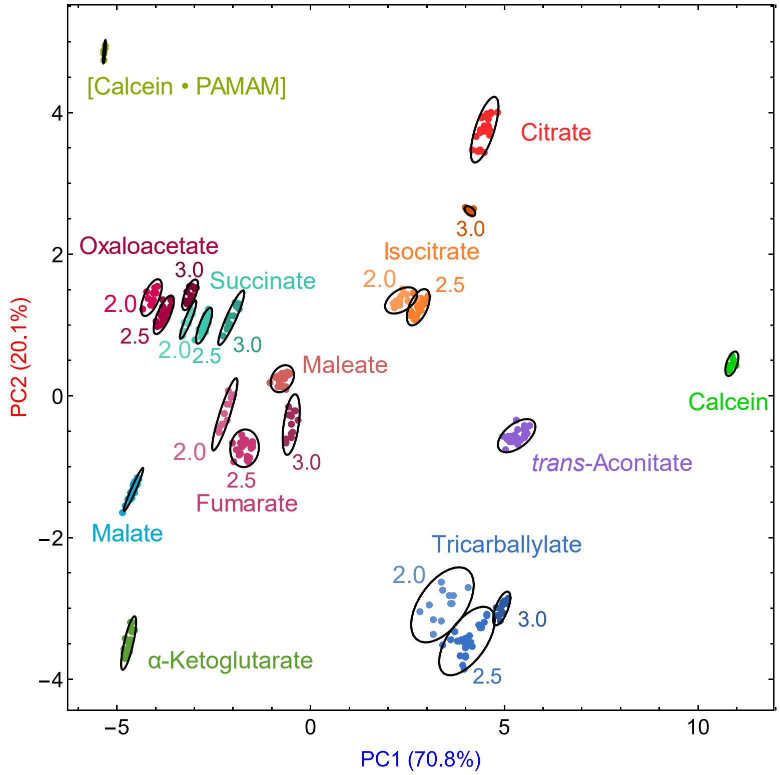

Figure S10. PCA 2D scores plot from the investigation of the effect of a small variation in concentration in the qualitative discrimination of the 10 carboxylate targets using the [calcein•PAMAM] complex sensor. The following anions appear over a range of concentrations (2.0 mM, 2.5 mM, 3.0 mM): oxaloacetate, succinate, isocitrate, fumarate, tricarballylate. Performed on a 384-well plate in 50 mM aqueous HEPES buffer at pH 7.4, T = 25 °C. [calcein] = 6.36 μM, [G5] = 2.13 μM.

Procedures for the determination of concentration and identity for unknown samples

The process for the determination of the concentration and identity of Unknowns **A**, **B** and **D** is detailed below (Unknown **C** is discussed in the main manuscript). Each sample was adjusted to pH 7.40 and a fluorescence anisotropy titration was carried out on a benchtop spectrofluorimeter. The results, plotted as the added volume of solution of unknown vs. anisotropy readings, were fit to standard concentration response curves for both tri and dicarboxylates. These curves, based on fluorescence anisotropy measurements for an average dicarboxylate / tricarboxylate, are shown as a line in the figures below; the points represent the experimental values, whose tentative concentration was adjusted to obtain the best fit to the standard curves.

1. <u>Preparation of probe samples:</u>

Fitting the points from Unknown **A** to the tricarboxylate standard curve established its tentative concentration at 54.3 mM (assuming a tricarboxylate). A first portion of Unknown **A** was therefore diluted 21.72 fold to 2.5 mM, 16 replicates were deposited on a 384-well plate (referred to as **A tri** in Figure 12 in the manuscript). On the other hand, fitting to the dicarboxylate standard curve determined a tentative concentration of 666 mM (assuming a dicarboxylate); a second portion of Unknown **A** was diluted 266.4 fold to 2.5 mM, 16 replicates were deposited on the same plate, and referred to as **A di** in Figure 12.

The same process was followed for Unknown **B**, for which tentative concentrations of 1.86 mM and 23.1 mM were calculated, as a tricarboxylate vs. dicarboxylate, respectively. The concentration of this anion as a tricarboxylate would have been slightly lower than the 2.5 mM normally used on the discrimination plate, but our previous study on the effect of small changes in concentration gave us confidence that a qualitative determination would still be successful, so a **B tri** sample was deposited as-is at 1.86 mM tentative concentration (similar to the 2.0 mM “low” concentration used in the concentration effect experiment). A **B di** sample was made by 9.24-fold dilution and added to the plate.

The same process was followed for Unknown **D**, obtaining tentative concentrations of 2.27 mM and 29.1 mM as a tricarboxylate and dicarboxylate, respectively. Once again, the 2.27 mM sample was used as-is as **D tri**; on the other hand, a portion of that sample was diluted 11.64-fold to generate **D di**. Both were added to the discrimination plate.

2. <u>Reference standards:</u>

True samples of the 10 carboxylates from our analyte panel were also added to the same plate as reference standards at 2.5 mM concentration in the same buffer. The plate also contained buffer blanks.

3. <u>Determination of unknown identity:</u>

As shown in Figure 12 in the main manuscript (reproduced below as **Figure S12** as well), **A tri** was found to overlap with *trans*-aconitate, while **A di** did not behave as any of the standard carboxylates. We therefore concluded that Unknown **A** was *trans*-aconitate, with a concentration of 54.3 mM; its true identity was indeed *trans*-aconitate, with a true concentration of 36.9 mM (47.2% error).

Similarly, **B di** was very similar to α-ketoglutarate, whereas **B tri** did not behave as any known carboxylates. We therefore determined Unknown **B** to be α-ketoglutarate, with a concentration of 23.1 mM; its true identity was indeed α-ketoglutarate, with a concentration of 25.4 mM (9.14% error).

Finally, **D tri** overlapped with the tricarballylate cluster, whereas **D** di could not be identified with of the carboxylate reference standards. Unknown **D** was found to be tricarballylate, with a concentration of 2.27 mM; its true identity was indeed tricarballylate, with a concentration of 3.05 mM (25.7% error).

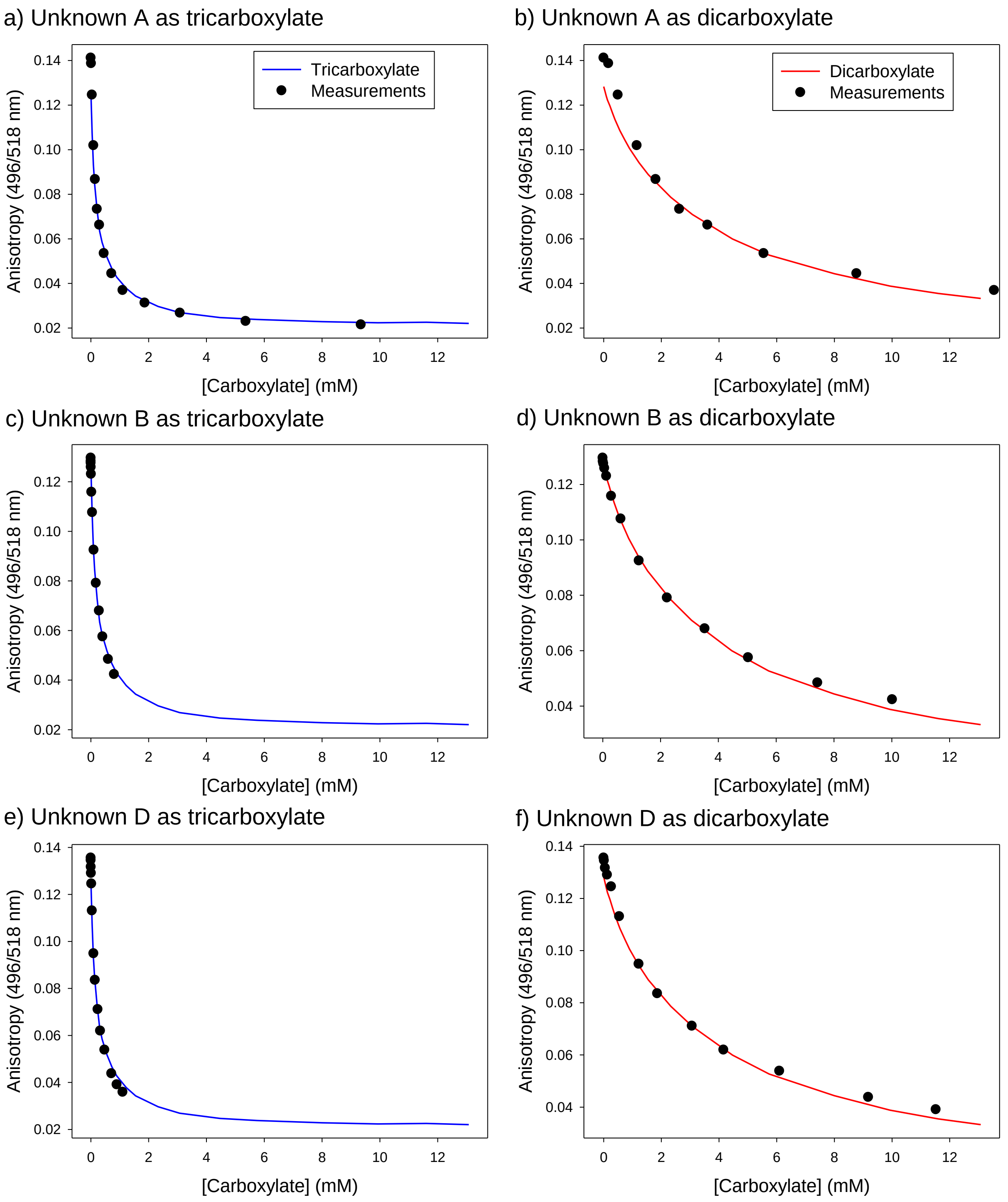

Figure S11. Concentration analysis of unknown: fit to the tricarboxylate (left) vs dicarboxylate (right) average concentration response curves. Top: Unknown **A**. Center: Unknown **B**. Bottom: Unknown **D**. Line = standard curve; black circles = experimental values. Performed in 50 mM aqueous HEPES buffer at pH 7.4, T = 25 °C. [calcein] = 6.36 μM, [G5] = 2.13 μM, excitation: 496 nm, emission: 518 nm.

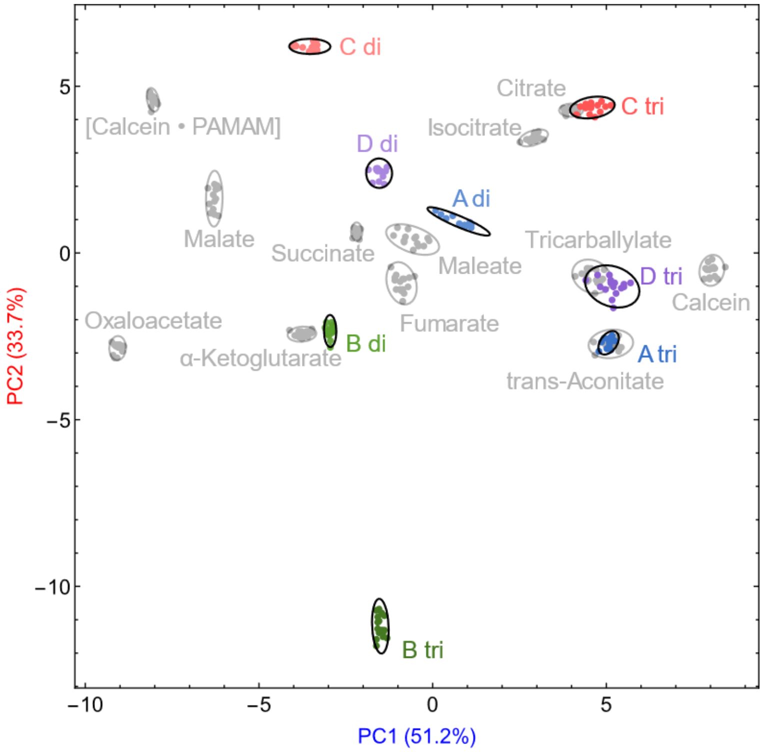

Figure S12. PCA scores plot for the simultaneous determination of identity and concentration of 4 unknown samples using the [calcein•PAMAM] complex sensor. In color: intermediate samples for each unknown; in gray: pure carboxylate reference samples. Each cluster represents a replicate of 16 samples. The experiment was performed on a 384-well plate in 50 mM HEPES aqueous buffer at pH 7.4, T = 25 °C. [calcein] = 6.36 μM, [G5] = 2.13 μM, [carboxylate reference samples] = 2.30 mM.

References

[1] W.M. Haynes, CRC Handbook of Chemistry and Physics. 91st Edition, Taylor & Francis Group2010, pp. 8-43, 48-46.
[2] A.-S.S. Hamad Elgazwy, The chemistry of tricarballylic acid (TCA), Curr. Org. Chem., 8 (2004) 1405-1423.
[3] I. Juranic, Z. Vitnik, Correlation of the physicochemical parameters of reactions with the theoretically calculated atomic charges, Society of Physical Chemists of Serbia, 2006, pp. 769-776.
[4] H. Chen, S. Laurent, S. Bedu, F. Ziarelli, H.-l. Chen, Y. Cheng, C.-C. Zhang, L. Peng, Studying the Signaling Role of 2-Oxoglutaric Acid Using Analogs that Mimic the Ketone and Ketal Forms of 2-Oxoglutaric Acid, Chem. Biol. (Cambridge, MA, U. S.), 13 (2006) 849-856.
[5] N.R. Clement, J.M. Gould, Pyranine (8-hydroxy-1,3,6-pyrenetrisulfonate) as a probe of internal aqueous hydrogen ion concentration in phospholipid vesicles, Biochemistry, 20 (1981) 1534-1538.
[6] M. Li, R.L. Harbron, J.V.M. Weaver, B.P. Binks, S. Mann, Electrostatically gated membrane permeability in inorganic protocells, Nat. Chem., 5 (2013) 529-536.
